# Gene expression changes in the salivary glands of *Anopheles coluzzii* elicited by *Plasmodium berghei* infection

**DOI:** 10.1186/s13071-015-1079-8

**Published:** 2015-09-23

**Authors:** Renato Pinheiro-Silva, Lara Borges, Luís Pedro Coelho, Alejandro Cabezas-Cruz, James J. Valdés, Virgílio do Rosário, José de la Fuente, Ana Domingos

**Affiliations:** Instituto de Higiene e Medicina Tropical (IHMT), Lisbon, Portugal; Unidade de Biofísica e Expressão Genética, Instituto de Medicina Molecular (IMM), Lisbon, Portugal; Center for Infection and Immunity of Lille (CIIL), Institut Pasteur de Lille, Lille, France; SaBio. Instituto de Investigación de Recursos Cinegéticos, IREC-CSIC-UCLM-JCCM, Ciudad Real, Spain; Institute of Parasitology, Biology Centre of the Academy of Sciences of the Czech Republic, České Budějovice, Czech Republic; Department of Veterinary Pathobiology, Center for Veterinary Health Sciences, Oklahoma State University, Stillwater, USA; Global Health and Tropical Medicine (GHMT), Instituto de Higiene e Medicina Tropical (IHMT), Lisbon, Portugal

**Keywords:** *Anopheles coluzzii*, Salivary glands, *Plasmodium berghei*, Sporozoite, RNA-seq, Glucose transporter, RNAi

## Abstract

**Background:**

Malaria is a devastating infectious disease caused by *Plasmodium* parasites transmitted through the bites of infected *Anopheles* mosquitoes. Salivary glands are the only mosquito tissue invaded by *Plasmodium* sporozoites, being a key stage for the effective parasite transmission, making the study of *Anopheles* sialome highly relevant.

**Methods:**

RNA-sequencing was used to compare differential gene expression in salivary glands of uninfected and *Plasmodium berghei*-infected *Anopheles coluzzii* mosquitoes. RNA-seq results were validated by quantitative RT-PCR. The transmembrane glucose transporter gene *AGAP007752* was selected for functional analysis by RNA interference. The effect of gene silencing on infection level was evaluated. The putative function and tertiary structure of the protein was assessed.

**Results:**

RNA-seq data showed that 2588 genes were differentially expressed in mosquitoes salivary glands in response to *P. berghei* infection, being 1578 upregulated and 1010 downregulated. Metabolism, Immunity, Replication/Transcription/Translation, Proteolysis and Transport were the mosquito gene functional classes more affected by parasite infection. Endopeptidase coding genes were the most abundant within the differentially expressed genes in infected salivary glands (*P* < 0.001). Based on its putative function and expression level, the transmembrane glucose transporter gene, *AGAP007752*, was selected for functional analysis by RNA interference. The results demonstrated that the number of sporozoites was 44.3 % lower in mosquitoes fed on infected mice after *AGAPP007752* gene knockdown when compared to control (*P* < 0.01).

**Conclusions:**

Our hypothesis is that the protein encoded by the gene *AGAPP007752* may play a role on *An. coluzzii* salivary glands infection by *Plasmodium* parasite*,* working as a sporozoite receptor and/or promoting a favorable environment for the capacity of sporozoites.

**Electronic supplementary material:**

The online version of this article (doi:10.1186/s13071-015-1079-8) contains supplementary material, which is available to authorized users.

## Background

Malaria is a mosquito-borne infectious disease caused by *Plasmodium* parasites, with 200 million cases estimated to occur around the world, each year. Human malaria is transmitted exclusively through the bites of infected *Anopheles* mosquitoes, being *Anopheles coluzzii* the main vector in Africa [1].

Transmission of *Plasmodium* parasites is initiated through the ingestion of gametocytes by female mosquitoes feeding on an infected individual. Male and female gametes produce, after fertilization, zygotes that differentiate into motile ookinetes and invade the midgut epithelium. Ookinetes differentiate into oocysts after emerging on the hemocoel side, mature and release thousands of sporozoites into the hemolymph. When infected mosquitoes bite an individual and release some of these sporozoites, the transmission cycle is completed [2].

Of all mosquito tissues and cell types that the sporozoites come in contact with, they only invade the salivary glands (SG) which, together with mosquito saliva, can be considered central to the interaction between parasite, vector and mammalian hosts [3]. The sporozoite attaches to the basal lamina and subsequently binds to the basolateral membrane of the epithelial cells. This attachment and invasion are facilitated by the interaction between sporozoite and SG surface molecules [4], meaning the invasion depends on parasite recognition of mosquito SG surface components [5]. The maturation of sporozoite in the SGs is a key stage for the effective transmission, increasing sporozoite ability to infect vertebrate hepatocytes [3].

Previous studies have demonstrated that carbohydrates receptors may have an important role on parasite-vector interaction [6–9]. Although the relation between oocysts and carbohydrates has been reported [6, 7], their interactions with SG remains unclear.

In mosquito, as well as in other arthropods, RNA-sequencing (RNA-seq) and RNA interference (RNAi) have been applied for *de novo* transcriptome assemblies and expression profiles obtained at a specific condition [10–12] and for the study of gene function [13–16].

The publication of genome sequences from several arthropod vector species [17, 18] combined with transcriptomics and proteomics analyses of their SG extracts [19–23] revealed new insights into the diversity of salivary components in these organisms. The SG transcriptome and proteome of *An. coluzzii* have been characterized, providing a full description of the salivary proteins in this species [24]. However, despite the fact that the genome of *An. coluzzii* has already been sequenced [17], a significant number of genes still do not have a putative function assigned and, although there are reports concerning the characterization of mosquito SG genes [25–27], little is known about SG protein-sporozoite interactions.

In the absence of a licensed malaria vaccine and facing an increase in parasite resistance to new combined drugs and to insecticides used for indoor spraying and in bed nets, the development of complementary measures for vector control are highly needed [28]. Preliminary results obtained in arthropod vectors with impact on both human and animal health revealed that protective antigens can be used for the development of new tools against both vectors and pathogens [29–33].

Herein, we report a RNA-seq analysis of differential gene expression in the salivary glands of *An. coluzzii* elicited by *P. berghei* infection. A catalogue of transcripts was produced and analyzed, providing a valuable platform for future research. Further, gene expression was experimentally validated. A SG membrane transporter gene exhibiting the highest expression level regarding the transport functional class was chosen for functional analysis and the effect of gene knockdown on malaria parasite levels was further evaluated. A three dimensional model was elaborated and discussed. These findings will improve our understanding of mosquito SG infection process, contributing to the development of new measures for malaria control.

## Methods

### Ethics statement

The maintenance and care of experimental animals was carried out in accordance to the Europe Directive 86/609/EEC and Portuguese law (Decreto-Lei 129/92) recommendations and protocol approved by the Divisão Geral de Alimentação e Veterinária (DGAV), Portugal, under Portaria 8 n°1005/92 from 23rd October. Authors directly involved with animal manipulation were licensed to conduct research using laboratory animals.

### Mosquito rearing

*An. coluzzii* s.s (*An. gambiae* molecular M form) of the Yaoundé strain mosquitoes were obtained from the Institute of Hygiene and Tropical Medicine insectary, reared at 27 °C, 70 % relative humidity under a 12 h light/dark photoperiod and fed *ad libitum* on a 10 % glucose solution. The adult female mosquitoes used in these experiments were aged between 3–5 days.

### *P. berghei* GFP infections

Mosquitos were infected with *P. berghei* parasites (strain ANKA), which constitutively express green fluorescent protein (GFP) [34]. Parasites were maintained by serial passage in 3- to 4-week-old female CD1 mice (*Mus musculus*) from frozen stocks. Parasitaemia were determined 2–3 days after passage using light microscopy by methanol fixation of air-dried blood smears and stained with 20 % (w/v) Giemsa solution. Mosquitos were allowed to feed on mice when parasitaemia was between 4 and 6 % and 4–5 exflagellations/field were observed. Female mosquitoes (100 per group) were fed on anesthetized mice during 40–45 min. Non-fed females were removed from the cage. Infected and control (fed on uninfected mice) mosquitoes were kept at 21 °C and 70 % humidity to allow parasite development. Midguts were dissected 8–9 days post-blood meal (PBM) to confirm the presence of oocysts, using fluorescent microscopy.

### RNA extraction

Mosquitos were cold anaesthetized on ice and placed onto a glass slide in phosphate-buffered saline (PBS). To obtain the SG free of other tissues, legs and the head were first pulled off using forceps, the thorax was pushed down and the connection between these tissues and the SG was cut using a needle-tip, under a stereoscopic microscope at 4X magnification (Motic SMZ-171B, China). SG were dissected 18–19 days PBM, collected in phosphate buffer containing DEPC (DEPC-PBS) before transferred to RNA later (Ambion, Austin, TX, USA) and frozen at −80 °C until utilization. Total RNA was extracted using the RNeasy kit (Qiagen, Inc., Valencia, California, USA). After extraction, the RNA samples were quantified and analyzed for purity on a ND-1000 Spectrophotometer (NanoDrop ND1000, Thermo Fisher Scientific, Whaltman, MA). Extracted RNA obtained from six pools of SG (three from infected mosquitoes and three from uninfected mosquitoes) was used for RNA-seq. To obtain the quantity needed to perform RNA-seq, the samples were pooled, resulting in one biological replicate of SG infected mosquitoes and one biological replicate of SG uninfected mosquitoes.

### RNA-seq library preparation and sequencing

RNA quality and integrity were checked on an Agilent 2100 Bioanalyzer Nano Chip (Agilent Technologies, Santa Clara, CA, USA). The RNA-seq library preparation and sequencing were performed according with Ayllón and co-workers (2015) [35], using total RNA and Illumina TruSeq™ RNA Sample Prep Kit v2 (Illumina, San Diego, CA, USA), following the manufacturer’s protocol.

The quality of the FASTQ sequences was enhanced by trimming off low-quality bases using the “Trim sequences” option of the CLC Genomics Workbench version 5.5.1 (CLC Bio, Cambridge, MA, USA). The quality-filtered sequence reads are used for further analysis with the CLC Genomics Workbench. First, an alignment against the *An. coluzzii* genome reference (ftp://ftp.ensemblgenomes.org/pub/metazoa/release-15/fasta/anopheles_gambiae/dna/) and calculation of the expression values was performed using the “RNA-seq” option. The comparison of expression values and statistical analysis was performed with the “Expression analysis” option. To normalize for the difference in number of mapped reads and transcript length, quality control was performed, comparing the overall distributions of the RPKM (reads per kilobase of exon model per Million mapped reads) expression between samples and groups [36]. Finally, samples were clustered into groups using a hierarchical clustering approach. The hierarchical structure was chosen because the Bayesian model-based approach reduces the bias caused by the absence of the biological replicates, increasing the precision of differentially expressed genes [37]. *P*-value calculation of the Z-test was based on the raw counts (total exon reads per gene). Genes were considered significantly differentially expressed if the *P*-value was below 0.05 and the fold - change greater than one standard deviations above or below the average fold change across all genes. To analyze the statistically represented gene classes or categories, the g: Profiler web server (http://biit.cs.ut.ee/gprofiler/) was used.

The gene selected for RNA interference assay was filtered using location predictors WoLF PSORT [38] and MultiLoc2 [39].

### Validation of RNA-seq data

A total of eighteen transcripts identified by RNA-seq as being differentially regulated and belonging to different functional classes were chosen based on fold-change value (Additional file 1: Table S1) to confirm RNA-seq results by quantitative real-time-Polymerase Chain Reaction (qPCR). Total RNA extracted from infected and non-infected *An. coluzzii* SG (100 per group) was used to synthesize cDNA.

The Primer 3 platform (http://bioinfo.ut.ee/primer3-0.4.0/) was used to design all primers (Additional file 2; Table S2). Gene expression was assessed by iQ™ SYBR® Green supermix for qPCR (Bio-Rad, Hercules, CA, USA) in a total volume of 20 μl, using the iCycler iQ™ (Bio-Rad, Hercules, CA, USA). PCR involved an initial denaturation at 95 °C for 10 min, 40 cycles of 10 sec at 95 °C, 45 sec at the appropriate annealing temperature for each set of primers (Additional file 2: Table S2). Fluorescence readings were taken at 62 °C after each cycle. A final extension at 72 °C for 5 min was completed before deriving a melting curve (60–95 °C) to determinate the quality of the amplicon. Relative expression results were normalized with *An. coluzzii* ribosomal protein S7 (Vectorbase: AGAP010592) as internal standard and analyzed by the 2 delta Ct (ΔΔCt) method [40]. Three biological replicates with independent preparations of total RNA were performed for each gene.

Pearson’s correlation was used to compare the results obtained in both RNA-seq and qPCR analyzes.

### Gene silencing assays

RNAi-mediated gene-silencing assays were performed to evaluate the effect of *AGAP007752* gene knockdown on *P. berghei*-infected *An. coluzzii* mosquitoes.

Specific primers containing T7 promoter sequences at the 5′-end were synthesized (Additional file 3: Table S3) and dsRNA produced using the MEGAscript T7 kit (Ambion, Austin, TX, USA), according to manufacturer’s instructions. An exogenous gene, mouse *beta-2microglobulin* (*β2M*) (GenBank: NM_009735) was used as control for the silencing experiments. The dsRNA was purified, diluted in sterile water to a concentration of 4 μg/ml and quality was assessed by spectrometry and agarose gel. For gene knockdown, we perform three experiments of 300 *P. berghei* infected female mosquitoes (three to five- days-old) for each gene. Fourteen days PBM, cold anesthetized mosquitoes were injected intrathoraxically with 69 nl (4 μg/ml) of dsRNA using a nano-injector (Nanoject, Drummond Scientific, Broomall, PA, USA). The control group was injected with ds*β2M*. Quantitative RT-PCR was used to verify the silencing effect. The unpaired two-tailed *t*-test was used to compare the different experimental groups.

### Sporozoite quantification

SG were dissected 3–4 days post injection (18–19 PBM) and pools of 15–20 SG per treatment were used to perform sporozoite quantification. Each SG pool was homogenized in a total volume of 100 μl of phosphate-buffered saline using a mini glass tissue homogenizer (Kontes Glass Co., Vineland, NJ, USA). Sporozoites were counted by light microscopy using a hemocytometer [25]. Sporozoites quantification was performed using three independent biological replicates.

### Phylogenetic analysis

Sequences were aligned with the MUSCLE (v3.7) program, configured for highest accuracy [41] and poorly aligned and unsupported regions were removed with Gblocks (v 0.91b) [42]. The maximum likelihood method, implemented in the PhyML programme (v3.0 aLRT), was used to reconstruct the phylogenetic tree [43, 44]. Reliability for internal branches was assessed using the bootstrapping method (1000 bootstrap replicates). For the Bayesian analysis we used MrBayes (v3.2.2) implementing a proportion of invariable sites and gamma-distributed rate variation across sites (I + G) with a WAG amino acid substitution model. Two independent runs were executed, each with four Markov Chain Monte Carlo (MCMC) of 50,000,000 generations, sampling every 1000th generation (this resulted in a StDev < 0.001). The two phylogenetic analyses agreed on tree topology; therefore, bootstrap values and posterior probabilities were included in a single tree. Graphical representation and editing of the phylogenetic tree was performed with TreeDyn (v 198.3) [45].

### Tertiary modelling and induced-fit ligand docking

To approximate an accurate tertiary model of the *An. coluzzii* protein, we used several protein structure prediction servers, namely FOLDpro [46], I-TASSER [47], 3D-Jigsaw, LOOPP [48], Rosetta [49], Phyre2 [50] and SwissModel [51]. To assess the quality of the output models and to choose the top candidates, we used Resprox [52], Qmean [53], ModFOLD [54]. A consensus from all three quality assessment servers agreed that the Rosetta models were the most adequate. We then manually inspected the top three Rosetta models to determine any unresolved secondary structures (i.e., α-helices). The top candidate was refined via minimization and optimization of the hydrogen-bond network by means of side chain sampling using the Schrodinger’s Maestro Protein Preparation Wizard [55]. Briefly, the Protein Preparation Wizard analyzes the structure to build a cluster of hydrogen-bonds and with the highest degree of sampling, the algorithm performs 100000 Monte Carlo orientations for each cluster. Based on the electrostatic and geometric scoring functions, the algorithm then determines an optimized structure.

## Results and discussion

### *An. coluzzii* SG transcriptome

RNA-seq is a quite recent technology that allows obtaining the whole transcriptome and absolute gene expression measurements, for defined conditions. RNA-seq has been used previously to obtain a depth annotation of *An. coluzzii* midgut and, for different purposes, in other mosquitoes as *An. funestus*, *An. albimanus* and *Ae. aegypti* [11, 12, 56–58].

For the characterization of *An. coluzzii* female SG transcriptome, we used RNA-seq to compare transcript abundance in *P. berghei-*infected and control uninfected SG. Three groups of 100 mosquitoes each, fed on infected and on uninfected healthy mice were used for RNA-seq analysis and further validation using qPCR. The oocysts counting on eighth day of infection showed that mosquitoes on infected group had 86–89 % infection rate; groups showing lower infection rates were rejected.

**Fig. 1:**
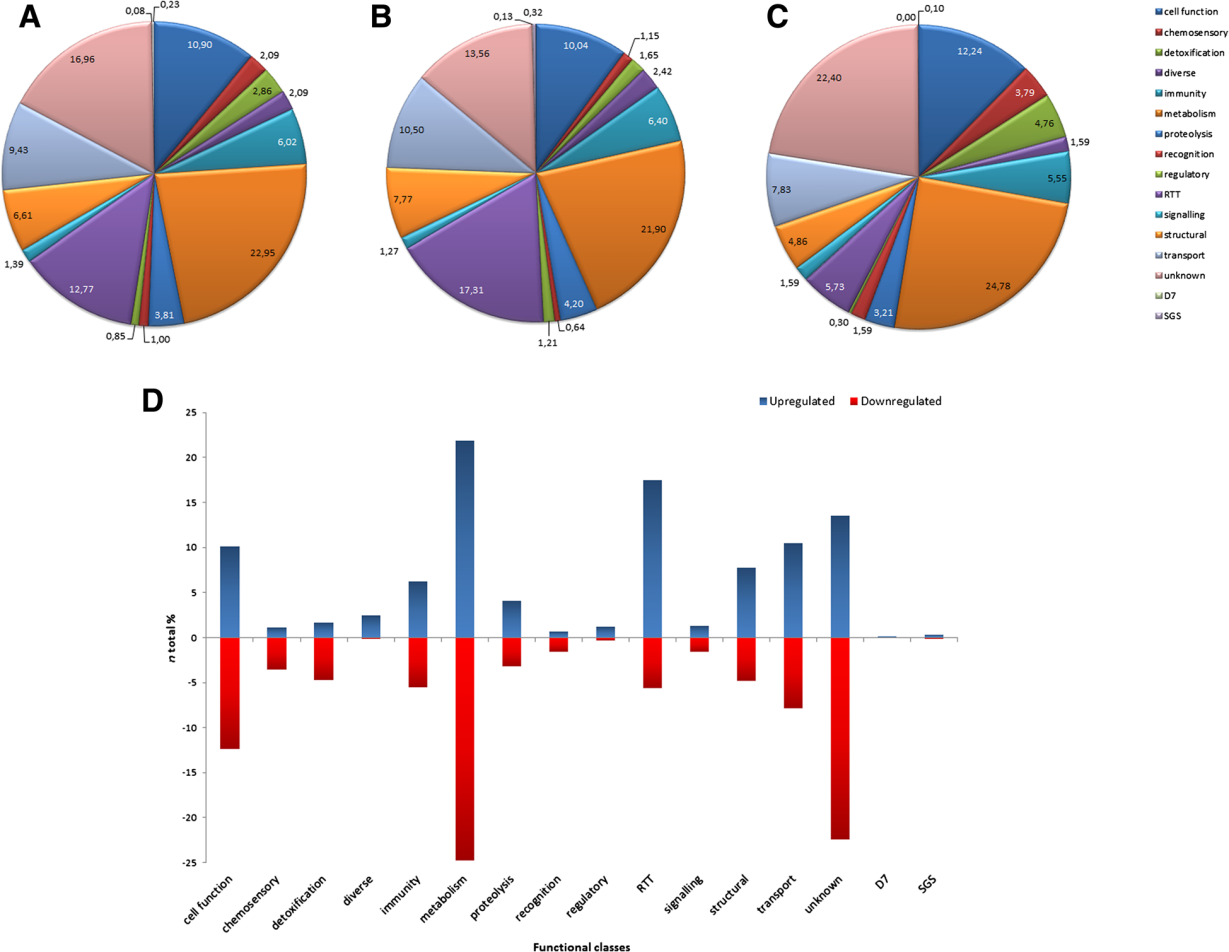
Transcriptional response in *An. coluzzii* SG infected with *P. berghei* based on Gene Ontology assignments. **a** Summary of the general distribution of differentially expressed genes (%). **b** Summary of the distribution of upregulated genes (%). **c** Summary of the distribution of downregulated genes (%). **d** Differentially expressed genes in *An. coluzzii* infected SG

The relative abundance of transcripts was analyzed. Of the total predicted transcriptome (15322 genes of *An. coluzzii,* according to version 72, ENSEMBL (http://www.ensembl.org/index.html), 12690 genes and 13611 transcripts were found in the SG transcriptome. Of them, 2588 genes were differentially expressed in response to *P. berghei* infection (Additional file 4: Table S4), being 1578 (61 %) upregulated and 1010 (39 %) downregulated (Fig. 1a-c). RNA-seq data are available in the ArrayExpress database (www.ebi.ac.uk/arrayexpress) under accession number E-MTAB-3415. These results indicates that RNA-seq is a high-throughput technique enabling to detect a high number of transcripts when compared with other approaches [59, 60], as microarrays that was recently used by Waisberg and co-workers [61], finding 43 genes differentially expressed.

To increase insight into SG transcriptome of *An. coluzzii* adult female mosquitos, differentially expressed products were functionally annotated using Gene Ontology (GO) terms description (Functional Class, Biological Process and Molecular Function) (Additional file 4: Table S4; Fig. 1a, d). Most of the genes were assigned as unknown function due to the lack of functional data. Quantitatively, genes belonging to Metabolism, Replication-Transcription-Translation (RTT) and Transport classes showed to be the most upregulated (Fig. 1b), whereas Metabolism, Cell Function and Transport were the most downregulated classes (Fig. 1c).

The expression of 18 genes identified as differentially expressed by RNA-seq (10 upregulated and 8 downregulated in response to infection) was analyzed by qPCR and RNA-seq results confirmed in 15 of them (Additional file 1: Table S1). Regression analysis between the two methods revealed a strong correlation between mRNA levels estimated by RNA-seq and qPCR (Pearson’s correlation coefficient r = 0.7957) (Additional file 5: Figure S1).

**Fig. 2:**
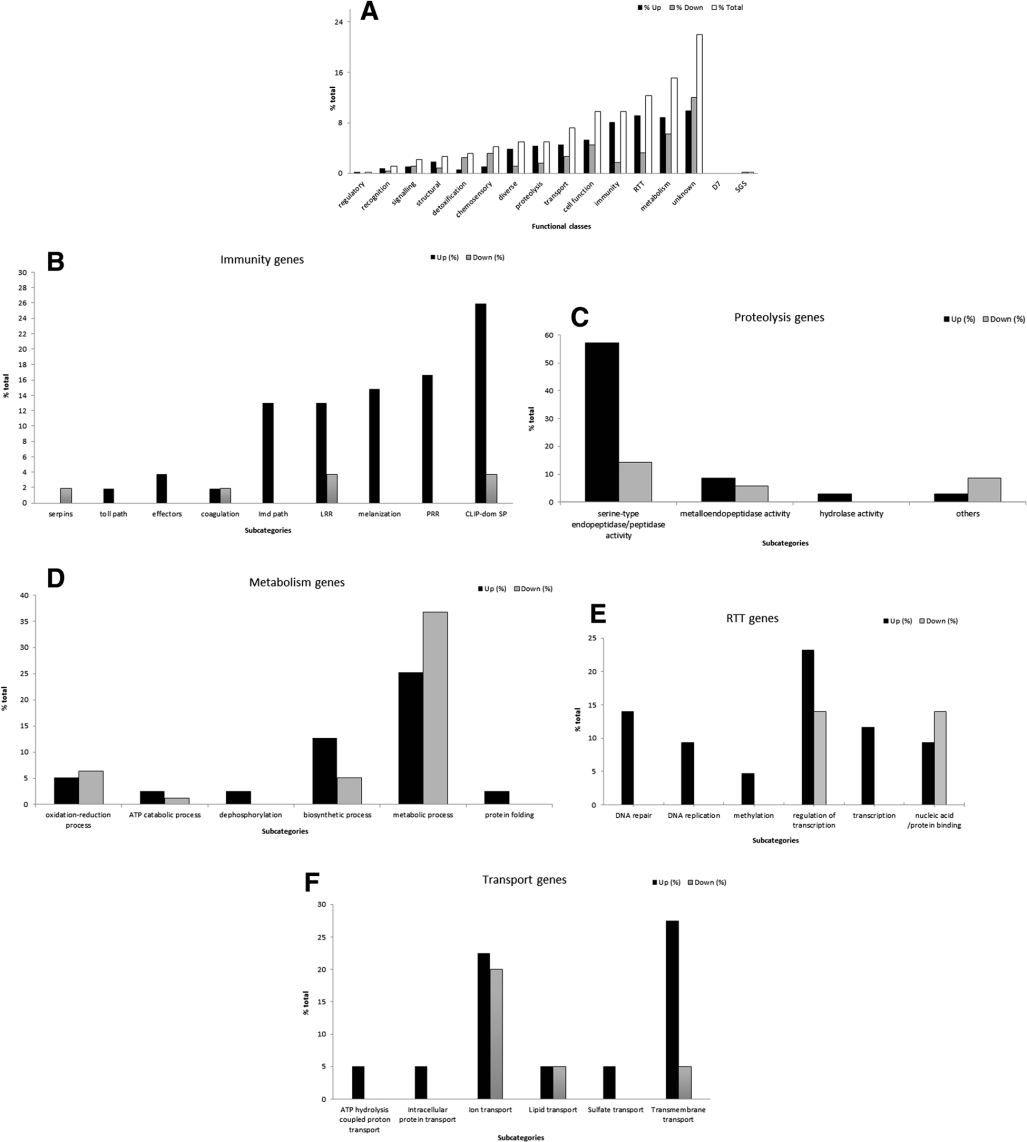
Differentially expressed genes in *An. coluzzii* infected SG based on Gene Ontology assignments. **a** General distribution of the differentially expressed genes (cut-off = 1.25). **b** Immunity gene class functional subcategories. **c** Proteolysis gene class functional and more representative functional subcategories. **d** Metabolism gene class functional subcategories. **e** RTT gene class and more representative functional subcategories. **f**—Transport gene class functional subcategories

As the majority of genes had a low fold-change value (75.6 % between 1.00 and 1.24), a cut-off of 1.25 was used (Fig. 2a) to obtain a working list including only genes with high fold-change values. For this cut-off, a total of 605 genes were obtained (Additional file 4: Table S4). The g: Profiler (http://biit.cs.ut.ee/gprofiler/) public web server for characterizing and manipulating gene lists from high-throughput genomics, was employed to identify gene classes or categories more represented within our RNA-seq data.

The molecular function “endopeptidase” was the category more statistically represented (P-value < 0.001) (Additional file 6: Table S5). Genes presenting an “endopeptidase activity” were classified as belonging to the Immunity and Proteolysis classes, as they participate in different pathways related to both classes.

Based on both g: Profiler and RNA-seq data, the predicted and more represented functional classes (Immunity, Proteolysis, Metabolism, RTT and Transport) were analyzed in detail.

### Immunity genes

Transcriptomic analysis using the fold - change cut-off 1.25 showed the highest number of differentially expressed genes among the immunity functional class from which 49 were upregulated and 06 downregulated. Within immunity, the subcategories classified according functional sub-class as Clip-Domain Serine Proteases (26 %), *PRR* (Pattern recognition receptors) (16.7 %), melanization (14.8 %), *LRR* (leucine-rich repeat) (13 %), and *Imd* pathway (13 %) were the most highly upregulated (Fig. 2b).

In a previous report, *An. funestus* transcriptome challenged with *Plasmodium* sp. was analyzed using BLAST to match results from RNA-seq and the *An. coluzzii* genome, showing *TEP*, *LRIM1* and Clip-Domain Serine Proteases (*SPCLIP1*) among the highest upregulated immunity genes [11]. *SPCLIP1* regulates the accumulation of TEP1 on malaria parasites and bacteria and can lead to distinct defense reactions including lysis and melanization of the pathogen [62].

Our data also showed that seven *LRR* genes were found upregulated; these proteins are related to the control of the complement-like protein TEP1 function having, as well, an important role on innate immune defense [63–65]. PRR are transcripts determinant for resistance to *Plasmodium* infection [66] and melanization, and also identified as playing an important role on antibacterial immune response on *An. coluzzii* [67]. Previous results highlighted the fact that the Imd pathway in *An. coluzzii* is part of an effort to limit the malaria transmission cycle, associating the Imd-directed immune response against *P. falciparum* [68] and showing that these genes are upregulated in response to *Plasmodium* infection [15]. Equivalent data were obtained in the present study, where all Imd-pathway genes (*AGAP010815*, *AGAP007035*, *AGAP007036*, *AGAP006348*, *AGAP007033* and *AGAP005693*) were found to be upregulated. These findings support the evidence that Imd pathway and *PRR* genes play a part in mosquito response to *Plasmodium* infection.

The gene *AGAP006421* was also found upregulated. It is considered a member of the antigen AG5 family of the SG mosquito transcripts [19], categorized as an immunity gene, but related with a family of secreted proteins with different functions [69].

### Proteolysis genes

GO analysis revealed that genes encoded proteins potentially related to proteolysis were more upregulated in mosquitoes SG infected with *P. berghei.* Twenty-five genes were found to be upregulated while 10 were downregulated, being the majority under the molecular function categorized as serine-type endopeptidases (57.1 %) (Fig. 2c).

The most upregulated transcript gene under this GO term was the *AGAP002596*, which corresponds to a metallopeptidase that have been associated with tissue invasion and infection by many pathogens [70].

It is known that this gene class is involved in blood meal and sugar digestion, namely in proteolytic events during blood-feeding on vertebrate hosts or in digestion of extracellular matrix components [71] and is, usually, upregulated in fed mosquito females [72]. Moreover, the biological significance of positive regulation may also be related with the role of these genes in immunity being part of the host defense system to limit spread of the parasite [73]. In addition, it have been showed that a salivary serine protease is related in Dengue transmission by *Aedes* mosquitoes [74].

### Metabolism genes

We found that genes putatively involved in metabolism functional class were highly transcribed in mosquito SG (Fig. 2d) in agreement with a previous transcriptomic analysis of *Ae. aegytpi* SG infected with Dengue virus suggesting parasite infection level is linked to physiological processes modifications [75].

An insulin-like peptide precursor and one gene classified as related with oxidation-reduction process were the most upregulated, while genes belonging to protein phosphorylation and lipid metabolism were less expressed. Insulin-like peptides (ILPs) regulate several biological processes as metabolism and immunity to infection. Previous reports conducted in several mosquito species have shown that ILPs secretion and action may be responsive in *Plasmodium*-infected females and potentially alter metabolism and innate immunity [76]. Our data show that gene expression changes in response to infection indicates the upregulation of genes associated with ubiquitin-dependent protein catabolic processes and with amino acid metabolism. The enrichment of functional terms such as ubiquitin-dependent proteasome was also denoted for insects facing dehydration stress [77]. Among the transcripts encoding metabolism, two, involved in nitric oxide biosynthetic process, were found to be upregulated. Nitric oxide synthase expression and nitric oxide increases in *An. coluzzii* and *An. stephensi* midgut after *Plasmodium* parasite infection [78, 79] limiting parasite development within the mosquito [80]. Further, within this GO term, transcripts linked to sugar metabolism were mostly upregulated whereas those associated with lipid metabolism were essentially downregulated, which may be connected to cell repair in response to infection and/or to the production of metabolites needed for sporozoites progress [59].

### RTT genes

Translational regulation allows cells to respond to stimuli and modify protein levels. For most of the genes, is not known if an increase on translation is directly related to defence reactions against *Plasmodium sp.* infection, but some reports evidence that translational regulation of gene expression in mosquito midguts has a profound impact on the anti-malaria responses [81].

As many as 43 genes, 31 upregulated and 12 downregulated (Fig. 2e) were found to be transcribed in SG of *An. coluzzii* belonging to the RTT class, which includes the second highest number of genes differentially expressed in response to parasite infection (Fig. 2a). Transcript genes linked to the biological processes DNA replication and DNA repair were exclusively upregulated, probably acting as cell defense from infection. Our data is in accordance to the transcriptome profile of *An. coluzzii* hemocytes upon *P. berghei* infection, also showing that the RTT class was also significantly upregulated [82]. RTT genes may regulate protein expression required for *Plasmodium*-SG interaction and thus, manipulated by the parasite to facilitate infection.

### Transport genes

Several transport transcripts (28 upregulated and 12 downregulated) were identified in SG (Fig. 2f). Among the subcategories described, we found that transmembrane transport transcripts were the most represented (27.5 %). The most abundant and upregulated transcript (AGAP007752), encoding a protein referred as EAA12343, was submitted to a location predictor analysis, confirming its inclusion into this subcategory. Although evidences suggest that this gene is not SG-specific, being preferentially expressed in Malpighian tubules from male mosquitoes [72], both RNA-seq and qPCR experiments showed a high upregulation expression in SG female mosquitoes during *Plasmodium* infection. Moreover, the GO annotation predict the function of this transcript as glucose transporter and, supported by findings that carbohydrates transporters may have an important role on *Plasmodium* transmission [6–9], we selected the *AGAP007752* gene for further analysis.

### Other functional classes

Among the genes selected to be analyzed by qPCR, the *HPX11* gene (*AGAP010899*), belonging to the detoxification functional class and, included in the biological process designated as response to oxidative stress, was one of the most highly downregulated (Additional file 1: Table S1). Transcription alteration of detoxification genes in response to bacteria and *Plasmodium* has been described [73]. It is possible that subexpression might be related with the presence of the parasite, as infection may induce stress response in mosquitoes [83], resulting in induced and or repressed genes [73]. During mosquito response to infection, active nitrogen and oxygen radicals are produced to contain *Plasmodium* infection [84]. These products may represent potential oxidative stress that can be enhanced or eliminated by detoxification enzymes.

Several genes with putative chemosensory molecular function as odorant binding proteins (OPB) were transcribed in *An. coluzzii* SG after infection. The expression of one of these genes, *OBP20,* was evaluated by RNA-seq and qPCR, showing to be downregulated in both assays (Additional file 1: Table S1). Olfaction plays a vital role in guiding mosquito behaviours and contributing to their ability to transmit pathogens but response mediated by chemosensory genes may vary according to the pathogen [73, 85].

In addition, two members of the D7 family, *AGAP008278* and *AGAP008279* (D7 long form L1 and L2, respectively) were found upregulated in SG. It is suggested that D7 SG proteins are distantly related with OBP, inhibiting hemostasis during hematophagy and therefore, facilitating blood feeding [86].

Six genes that codify five members of the salivary gland surface (SGS) were differentially expressed (*AGAP000548* (SGS1b), *AGAP000150* (SGS6), *AGAP008215* and *AGAP008216* (SGS7), *AGAP010647* (SGS8) and *AGAP003841* (SGS10)). These transcripts codify a family of immunogenic mosquito SG proteins involved during blood feeding and also parasite infection [87, 88].

### *AGAP007752* silencing experiments

Considering that membrane transporter proteins of *Anopheles* sp. SG have an important role on *Plasmodium* transmission, we select the *AGAP007752* SG gene coding to a membrane transport protein to perform RNAi-mediated gene silencing. This gene showed to be the most upregulated within the transport functional class regarding RNA-seq analysis (Additional file 4: Table S4), showing to be as well upregulated in qPCR experiments (Additional file 1: Table S1).

The *AGAP007752* gene was annotated as predicted coding for a protein with transmembrane transporter activity (according to Gene Ontology annotation: http://www.ebi.ac.uk/QuickGO/GTerm?id=GO:0055085). Protein-protein interaction prediction analysis using the platform STRING (string-db.org) showed that this target interacts with only four proteins (AGAP000128, AGAP000220, AGAP003039 and AGAP006360). Two of them, AGPA006360 and AGAP000220 were found on RNA-seq results but they showed no similarity function. The low number of interactions and no similar function associated suggests this target as a good candidate for further analysis. Thus, to characterize the function of this gene in *Plasmodium* sporozoite SG invasion, putative function analysis and RNA interference (RNAi)-mediated gene knockdown was carried out in infected and uninfected mosquitoes.

**Fig. 3:**
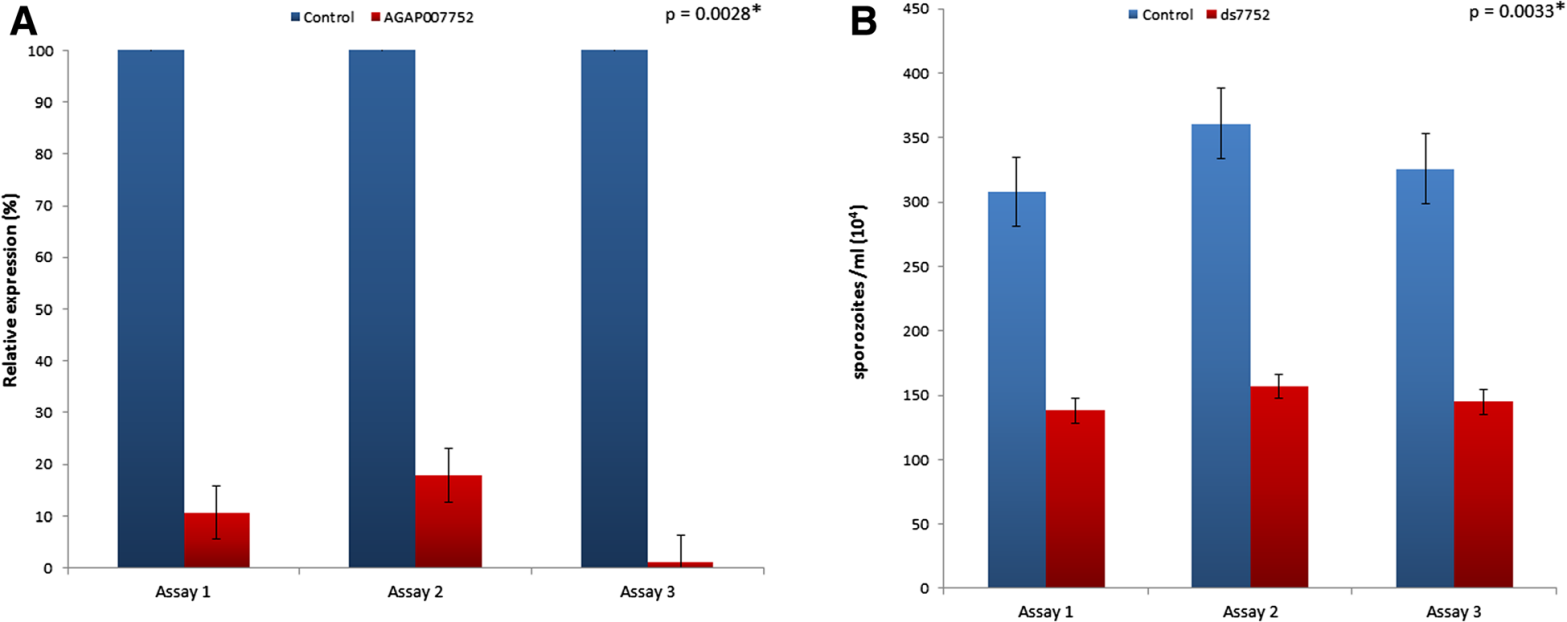
Effect of *AGAP007752* silencing on *An. coluzzii* SG infection by *P. berghei*. Infected mosquitoes were injected with dsRNA 14 days PMB and SG extracted 4–5 days later to determine sporozoite numbers and mRNA levels by qPCR. **a** Normalized *AGAP007752* mRNA levels (9.9 + 5.1) were expressed in arbitrary units and compared between groups using the unpaired two-tailed *t*-test (*P < 0.01; *N* = 3). **b** Effect of *AGAP007752* silencing on the number of *P. berghei* sporozoites present in the SG of mosquitoes injected with dsRNA*7752* (*AGAP007752*) when compared to controls (146.7 + 9.6; unpaired two-tailed *t*-test *P < 0.01; *N* = 3)

Performing three independent RNAi assays, we found a significant 99 %, 82 % and 89 % (146.7 + 9.6) (unpaired two-tailed *t*-test, *P* < 0.01) reduction on endogenous mRNA levels (Fig. 3a). *AGAP007752* knockdown significantly reduced the number of sporozoites present in the SG 18 days post-infection by 45 %, 43 % and 44 % (9.9 + 5.1) (unpaired two-tailed *t*-test, *P* < 0.01) when compared to controls (Fig. 3b).

Glucose transporters are a wide group of membrane proteins essential for transport and metabolism of glucose in cells of diverse organisms from microbes to humans [89]. The EAA12343 was categorized as glucose transporter and, as other proteins from this family, is likely to be glycosylated [90]. Previous reports have suggests that *Plasmodium* spp. propagation in mosquitoes consumes vector nutrients [8] and specific glycosylated proteins might function as parasite receptors on the basal lamina, of the distal lateral and the medial lobes of the SG [6, 91]. Furthermore, the invasion of *P. gallinaceum* sporozoites into the SG of *Aedes aegypti* is blocked by a carbohydrate binding protein or lectin [7], confirming the role of glycosylated proteins as receptors for malaria sporozoite-SG interaction.

In accordance to our data, it was shown that the knockdown of the sugar transporter trehalose (*Ag*TreT1) significantly reduced parasite oocysts in the midguts of *An. gambiae* infected with *P. falciparum*; threalose is the predominant sugar in mosquito hemolymph decreasing after *Plasmodium* infection [8]. *Ag*TreT1 showed to be critical to maintain hemolymph trehalose concentration and a positive mediator for parasite growth and propagation during the oocyst stage. Sporozoite may scavenger and metabolizes trehalose directly or trehalose may be hydrolyzed to glucose by either the vector or the sporozoite [8]. Rosinski-Chupin and co-workers [59] found several genes involved in energy and glucose metabolism upregulated in SG infected by *Plasmodium* sp., assuming that this alterations may be favorable for the sporozoite maturation and/or transmission to the vertebrate host. According with these findings, it is likely that the EAA12343 protein might act as a sporozoite receptor for entry in the SG and/or have part on the maintenance of parasite optimal conditions, as glucose level, supplying energy for the vertebrate infection.

Other proteins have been described as related to *Plasmodium* infection on *Anopheles* spp.. For instance, it have been demonstrate that Saglin (a protein found in *Anopheles* SG) and their interaction with TRAP (a surface protein present in *Plasmodium* parasites) is essential for parasite invasion in SG [2]. Another protein, AgESP, was found in both midgut and SG and described as having an important role on *Plasmodium* infection, reducing midgut invasion and the number of sporozoites, after silencing. Some proteins, however, show a protective effect on *Plasmodium* infection. This is the case of the SRPN6, a marker of *Plasmodium* infection in *An. coluzzii* SG, as once the gene is silenced, the number of sporozoites increases in SG [25]. Further, the passage through the SG is necessary for sporozoite capacity, as the complete acquisition of gliding locomotion and cell traversal [92].

### *An. coluzzii* protein putative function

**Fig. 4:**
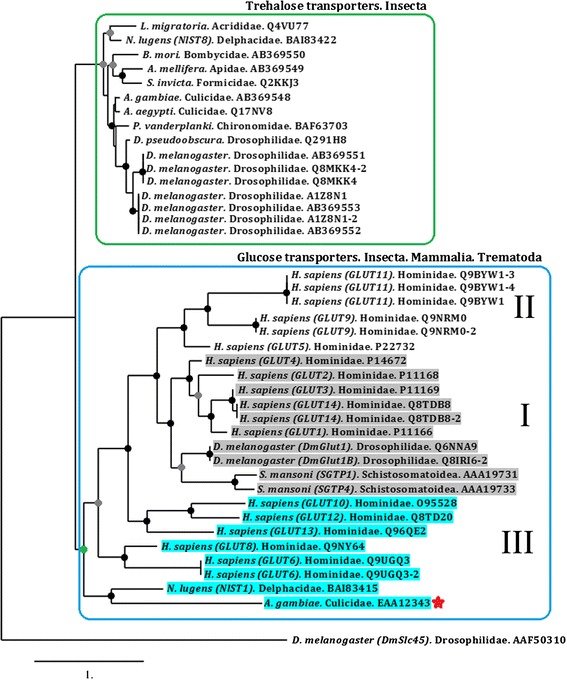
Phylogenetic tree of trehalose and glucose transporters family. Maximum likelihood (ML) and MrBayes (MB) phylogenetic analysis for trehalose (green box) and glucose transporters (blue box) were constructed and showed similar phylogenetic topology. Coloured circles represent the statistical support for each clade. Nodes with black circles indicate ≥ 90 % of bootstrap and ≥ 0.9 of posterior probability, grey circles indicate ≥ 60 % bootstrap and ≥ 0.9 of posterior probability and green circles indicate 52 % bootstrap and 0.5 posterior probability. Nodes without circles have ≤ 50 % bootstrap and ≤ 0.5 posterior probability. The sequence names were written following the code: species name, taxonomic family and GenBank accession number. The sucrose transporter from *D. melanogaster* DmSlc45 (accession number AAF50310) was used as an outgroup. *An. coluzzii AGAP007752* is indicated by a red star

Classifying the putative function of the *An. coluzzii* protein coded by the *AGAP007752* gene as a sugar transporter was done by performing five PSI-BLAST [93] interactions using the default settings—a pfam database search [94] confirmed this classification. The top hits were from insect trehalose and glucose transporters (E value 3e-38). It was previously shown that insects glucose transporters cluster with several organisms, including mammalian glucose transporters and not with insecta trehalose transporters. suggesting a function related to phylogenetic clustering [95]. Maximum likelihood and Bayesian phylogenetic analyses with threalose and glucose transporters from members of metazoa (Mammalia, Insecta and Trematoda) show that *An. coluzzii AGAP007752* protein (named as EAA12343) (Fig. 4, red star) cluster together with glucose transporters from the three metazoan clades (Fig. 4, blue box) and not with the trehalose transporters from insecta (Fig. 4, green box) (Additional file 7: Figure S2, Additional file 8: Figure S3 and Additional file 9: File S1), in agreement with previous findings [95]. The GLUT/SLC2A glucose transporter family in humans has been categorized into three classes: class 1 comprises GLUT1 to GLUT4 and GLUT14-L; class 2 comprises GLUT5, GLUT7, GLUT9, and GLUT11 and class 3 comprises GLUT6, GLUT8, GLUT10 and GLUT12 [96–98]. We found that EAA12343 cluster together with an insecta glucose transporter (NlST1) that was previously characterized [99] and both of them cluster together with class III human glucose transporters (Fig. 4, blue box).

**Fig. 5:**
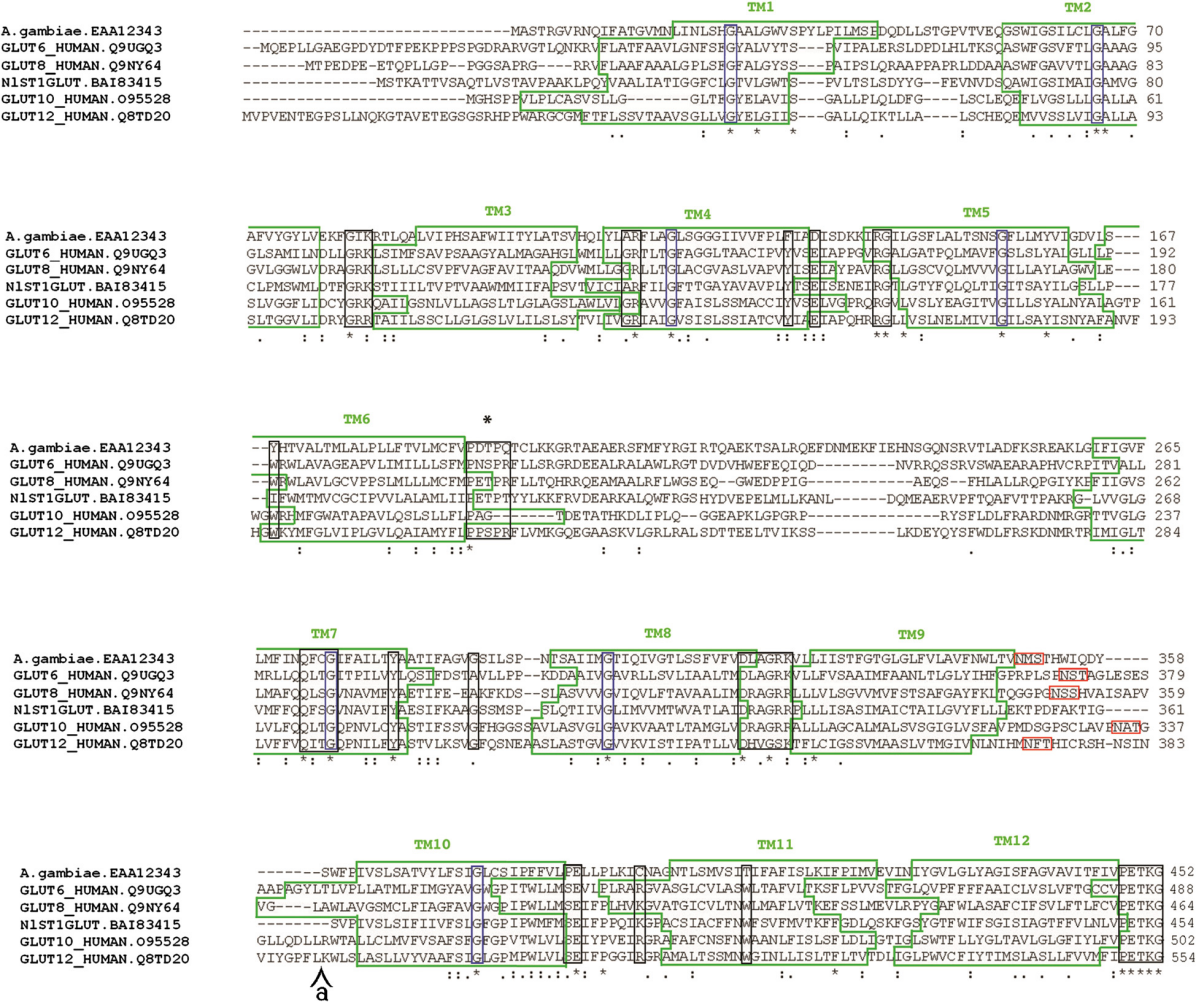
Alignment of members of glucose transporters class III. Class III glucose transporters were aligned using Clustalw. Relevant motifs conserved among class III glucose transporters and *An. coluzzii* EAA12343 are shown. Annotation of relevant motifs was done using previous reports [95, 96, 98]. Transmembrane domains (TM) are indicated (green boxes), conserved glycine (G) residues (blue boxes), N-linked oligosaccharide site (red boxes) and “PXXPR” motiff (black box with asterisk on top). Other residues and motiff conserved among class III glucose transporters are also shown (black boxes). Insertions of 60 and 68 amino acids in the sequences GLUT10 and GLUT12 respectively were deleted from the alignment, the position is indicate (a). Transmembrane domains were predicted using TMHMM 2.0 [100]

To confirm our phylogenetic classification, we performed a multiple amino acid sequence alignment of glucose transporters class III (NlST1GLUT/GLUT8/GLUT6/GLUT10/GLUT12). The alignment shows that the *An. coluzzii* glucose transporter identified in this study share important functional domains with other members of this class of glucose transporters (Fig. 5). For example, a common structural feature of the GLUT/SLC2 family members is the presence of 12 transmembrane domains (TM) and a unique N-linked oligosaccharide side-chain present in a large extracellular loop either to the N-terminus (classes I and II) or to the C-terminus of the protein (class III) (Fig. 5, red boxes). The conserved glycine (G) residues in the transmembrane domains “(TM) 1, 4, 5, 7, 8, and 10” among class III glucose transporters (Fig. 5, blue boxes) are thought to be critical in stabilizing the structure of GLUT/SLC2A [96]. Another difference among glucose transporter classes is the proline-containing motif between TM6 and TM7. Classes I and II possess residues PETPR/PESPR, respectively [95], while class III possess “PXXPR” [95] (Fig. 5, asterisks above black box). Finally, the presence of a dileucine motif in the amino-terminal tail of some members of class III glucose transporters has been previosuly reported [96]. In our case, even when leucines residues were found in the amino-terminus of both NlST1 and EAA12343, no dileucine motif was present.

**Fig. 6:**
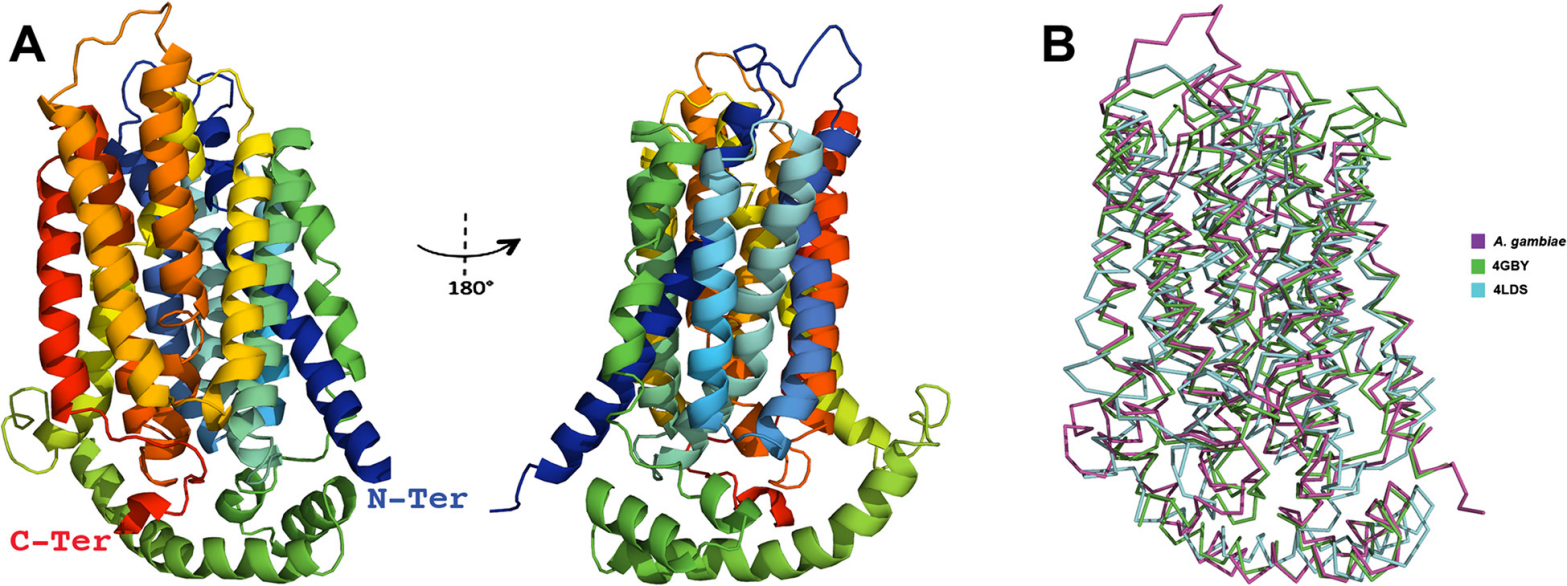
Tertiary predicted structure of *An. coluzzii* EAA12343 protein. The tertiary predicted structure of *An. coluzzii* EAA12343 (**a**), rotated 180° around the y-axis, is color coded from the amino-terminus (blue) to the carboxyl-terminus (red). The superimposition of the C-backbone (**b**) was performed using the protein structural alignment tool of Schrodinger’s Maestro program [101]. The sugar transporters from *Escherichia coli* (PDB: 4GBY) and *Staphylococcus epidermidis* (PDB: 4LDS) were used for comparison

The predicted tertiary structure of EAA12343 is depicted in Fig. 6a and clearly shows the 12 TM domains common among membrane bound transporters (as indicated by the alignment in Fig. 5). There are several GLUT/SLC2A crystal structures available, however, not all classes have been resolved. Regardless of the different classes, the overall structure GLUT/SLC2A is highly conserved. In Fig. 6b we demonstrate the conservative nature of the structural backbone of EAA12343 compared with two other sugar transporters for which crystal structures are available. The overall root mean square deviation of the superimposed structures in Fig. 6b is < 3.5 Å, indicating that the tertiary model of EAA12343 is adequate for further computational analyses.

## Conclusions

This is the first transcriptome analysis of *An. coluzzii* SG infected with *P. berghei* by RNA-seq, producing a catalogue of SG genes differentially expressed in response to infection. Among the differentially expressed genes, the sugar transporter *AGAP007752* gene was the most upregulated after *P. berghei* infection and the RNAi-mediated gene knockdown data suggests that *AGAP007752* encoded a protein that may be important to malaria transmission. Thus, we suggest that this protein may have an analogous role to other sugar transporters as a sporozoite receptor and/or in the maintenance of the optimal conditions for parasite egress from salivary glands.

Further analyses are being conducted to go deeper into the role of EAA12343 protein on *P. berghei* transmission, as protein location in SG and its impact on mosquito physiology after gene-silencing.

Our set of results provide a valuable resource for future studies in this crucial malaria vector. The improvement on the understanding of salivary gland gene expression and function will contribute for the progress on malaria control actions.

## Additional files

Additional file 1: Table S1. mRNA levels for selected genes as obtained by RNA-seq and qPCR. (DOCX 24 kb) Additional file 2: Table S2. List of primers used for qPCR gene expression confirmation. (DOCX 27 kb) Additional file 3: Table S3. List of primers used for double stranded RNA synthesis. (DOCX 17 kb) Additional file 4: Table S4. List of genes expressed in the female salivary glands of *An. coluzzii* of the group infected with *P. berghei* vs. uninfected. The excel file has two sheets: Sheet 1 list the genes that are expressed using one standard deviations above or below the average fold change across all genes. Sheet 2 lists the genes that are expressed using cut-off 1.25. The gene list is further sorted into different functional groups, according to the predicted function of the genes. Functional sub-classes, Biological process and Molecular Functional information were obtained according to Gene Ontology annotation, Ensembl metazoa (metazoa.ensembl.org) and Uniprot (uniprot.org). (XLSX 338 kb) Additional file 5: Figure S1. Comparison of mRNA levels determined by RNA-seq and qPCR. The ratios of mRNA levels found in RNA-seq were compared with the corresponding values obtained using qPCR. A strong correlation was observed between the two data sets as demonstrated by the Pearson’s correlation coefficient (*P* = 0.7957). (TIFF 8 kb) Additional file 6: Table S5. Results from g: Profiler analysis. (XLS 97 kb) Additional file 7: Figure S2. MrBayes phylogenetic tree of trehalose and glucose transporters family. Maximum likelihood (ML) phylogenetic analysis for trehalose and glucose transporters. Bootstraps values are shown. (PDF 17 kb) Additional file 8: Figure S3. Maximum likelihood phylogenetic tree of trehalose and glucose transporters family. Maximum likelihood (ML) phylogenetic analysis for trehalose and glucose transporters. Bootstraps values are shown. (PDF 5 kb) Additional file 9:
**File S1.** Alignment for trehalose and glucose transporters used in the phylogenetic analysis. (DOC 113 kb)
