# Supplementary material for: Gene expression changes in the salivary glands of Anopheles coluzzii elicited by Plasmodium berghei infection
Source: Parasit Vectors. 2015 Sep 23;8:485. doi: 10.1186/s13071-015-1079-8 (PMC4580310; doi:10.1186/s13071-015-1079-8)
Supplement: Additional file 1: Table S1. — mRNA levels for selected genes as obtained by RNA-seq and qPCR. (DOCX 24 kb) [file 13071_2015_1079_MOESM1_ESM.docx]

**Table S1.** **mRNA levels for selected genes as obtained by RNA-seq and qPCR (infected to uninfected log fold-change).**

| **Gene ID** | **Accession number** | **RNA-seq** | **qPCR** |
| --- | --- | --- | --- |
| 7752 | AGAP007752-RA | 2.18 | 1.83 |
| DEFI | AGAP011294-RA | -1.05 | -0.47 |
| LRIM17 | AGAP005693-RA | 1.25 | 0.80 |
| 5796 | AGAP005796-RA | 1.48 | 0.60 |
| SRPN12 | AGAP001375-RA | -2.63 | -0.66 |
| CTLGA2 | AGAP006430-RA | 1.55 | 0.90 |
| LYSC2 | AGAP007343-RA | 3.36 | 5.40 |
| 11654 | AGAP011654-RA | 3.32 | 5.40 |
| OBP20 | AGAP005208-RA | -2.03 | -1.32 |
| 10772 | AGAP010772-RA | 3.81 | 4.03 |
| 7976 | AGAP007976-RA | -1.71 | -0.64 |
| HPX11 | AGAP010799-RA | -2.51 | -1.40 |
| 6020 | AGAP006020-RA | -2.06 | -2.00 |
| TEP1 | AGAP010815-PA | 1.83 | 1.60 |
| 7782 | AGAP007782-RA | 3.98 | 0.70 |
| 298 | AGAP000298-RA | -2.15 | 0.26 |
| CYP6Z1 | AGAP008219-RA | -1.25 | 1.32 |
| 4170 | AGAP004170-RA | -1.34 | 0.03 |
