# Supplementary material for: Gene expression changes in the salivary glands of Anopheles coluzzii elicited by Plasmodium berghei infection
Source: Parasit Vectors. 2015 Sep 23;8:485. doi: 10.1186/s13071-015-1079-8 (PMC4580310; doi:10.1186/s13071-015-1079-8)
Supplement: Additional file 2: Table S2. — List of primers used for qPCR gene expression confirmation. (DOCX 27 kb) [file 13071_2015_1079_MOESM2_ESM.docx]

**Table S2. List of primers used for qPCR gene expression confirmation.**

| **Gene ID** | **Forward sequence** | **Reverse sequence** | **Annealing** |
| --- | --- | --- | --- |
| Ribosomal protein S | 5’ CAT TCT GCC CAA ACC GAT GCG 3’ | 5’ CGG GAA TAC CAG ATC CTC CAG G 3’ | 62°C° |
| AGAP007752 | 5’ TTG GTG CAC TCT TTG GAG CA 3’ | 5’ GAT GGA CGG AAG TGG CAA GA 3’ | 57°C |
| AGAP11294 | 5’ GGA GAA CTA TCG GGC CAA G 3’ | GAT ACA GTG AGC GGC ACA AA 3’ | 55,5°C |
| AGAP005693 | 5’ CAC AAT GGA GCT GGA CCG TA 3’ | 5’ TGC AAA CTG GAC GGT GGA TA 3’ | 58°C |
| AGAP005796 | 5’ CGG CAC GAT CCT AAC TTT TC 3’ | 5’ ATC GAT CAT GTG CTG CTG TT 3’ | 57°C |
| AGAP001375 | 5’ TGG TGG TCG AGG AGG AGA T 3’ | 5’ AGG GCC GAC TTG ATC TTC AG 3’ | 57,5°C |
| AGAP006430 | 5’ CCA ATC TCA CCA CTC ACA ACG 3’ | 5’ CCC GCT CGT GGA TGA TTC T 3’ | 56,5°C |
| LYSC2 | 5’ GGA CCA CTG CAG AGG AAA AG 3’ | 5’ ATG ACG ACA GGC TAC AGC AA 3’ | 56,5°C |
| CYP6Z1 | 5’ CAG TAT CTG GAA CGC TTG GC 3’ | 5’ GTG CAG ACA GTT TGC TTG GA 3’ | 56°C |
| AGAP004170 | 5’ CGA ATG TGT TCG AGT TCC AG 3’ | 5’ GAG ACG ATG TCG TTG CTG TC 3’ | 55°C |
| AGAP010772 | 5’ ACG ATC ATC ATA ACG CCA CA 3’ | 5’ GAT TCC ATT CCG TGC AGT TT 3’ | 56°C |
| AGAP011654 | 5’ TAA GAA CGG GAC CAC CTT TG 3’ | 5’ TAC TAT CAC GCA CCC CTT CC 3’ | 56,5°C |
| AGAP007976 | 5’ GCG GTG CTG AAG ATC AAG TA 3’ | 5’ TCA CGT CAA AGT CCA TCT CG 3’ | 55°C |
| AGAP007882 | 5’ GTC CCG CCA TCT GTG ATA AG 3’ | 5’ CAG TAG TGC GTG ACG GGA TA 3’ | 57°C |
| TEP1 | 5’ GTC CAG CCG TTC TAC ATC GT 3’ | 5’ GTA CAG CGT CAC ATC GGC TA 3’ | 57°C |
| HPX11 | 5’ CAC TGT TTG CAC GGT TGA TT 3’ | 5’ GCC CTC ATC GAA AAC TTC AA 3’ | 56°C |
| OBP20 | 5’ AAA TGA TAC GGT CGG TTT GC 3’ | 5’ TTT CCA TCA CGC AGT TTA CG 3’ | 56°C |
| AGAP006020 | 5’ GGC GAG ATG CAT TG TTC 3’ | 5’ CGG TAC ACC GGA TCC TTA AA 3’ | 55°C |
| AGAP000298 | 5’ AAG TGT TTC GAG CTG GGTG CT 3’ | 5’ CAC AGC AGC AGT ATC GTG GT 3’ | 58°C |
