# Supplementary material for: Gene expression changes in the salivary glands of Anopheles coluzzii elicited by Plasmodium berghei infection
Source: Parasit Vectors. 2015 Sep 23;8:485. doi: 10.1186/s13071-015-1079-8 (PMC4580310; doi:10.1186/s13071-015-1079-8)
Supplement: Additional file 3: Table S3. — List of primers used for double stranded RNA synthesis. (DOCX 17 kb) [file 13071_2015_1079_MOESM3_ESM.docx]

**Table S3.** **List of primers used for double stranded RNA synthesis.**

| **Gene name** | | **Forward sequence** | **Reverse sequence** | | **Annealing** | |
| --- | --- | --- | --- | --- | --- | --- |
|  | ds7752 | 5’TAATACGACTCACTATAGGGAGAAGTCTCGCGAAGCAAAGC-3’ | | 5’TAATACGACTCACTATAGGGAGAAGGGGTAATAATTCTGGT-3’ | | 63°C |
| dsβ2M | | 5’TAATACGACTACCTATAGGGAGACACCCCCACTGAGACTGATACA-3’ | | 5’TAATACGACTACCTATAGGGAGACACCCCCACTGAGACTGATACA-3’ | | 64°C |
| Underlined sequence belongs to T7 promoter. | | | | | | |
