# Supplementary figures and images for: Gene expression changes in the salivary glands of Anopheles coluzzii elicited by Plasmodium berghei infection
Source: Parasit Vectors. 2015 Sep 23;8:485. doi: 10.1186/s13071-015-1079-8 (PMC4580310; doi:10.1186/s13071-015-1079-8)

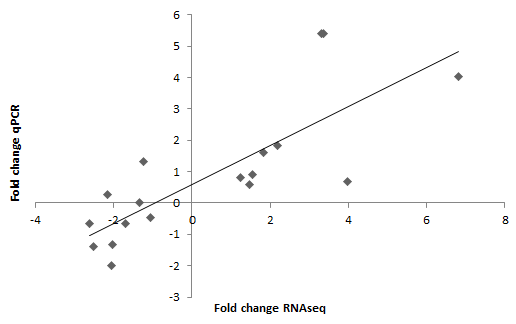

Supplement: Additional file 5: Figure S1. — Comparison of mRNA levels determined by RNA-seq and qPCR. The ratios of mRNA levels found in RNA-seq were compared with the corresponding values obtained using qPCR. A strong correlation was observed between the two data sets as demonstrated by the Pearson’s correlation coefficient (P = 0.7957). (TIFF 8 kb) [file 13071_2015_1079_MOESM5_ESM.tif]

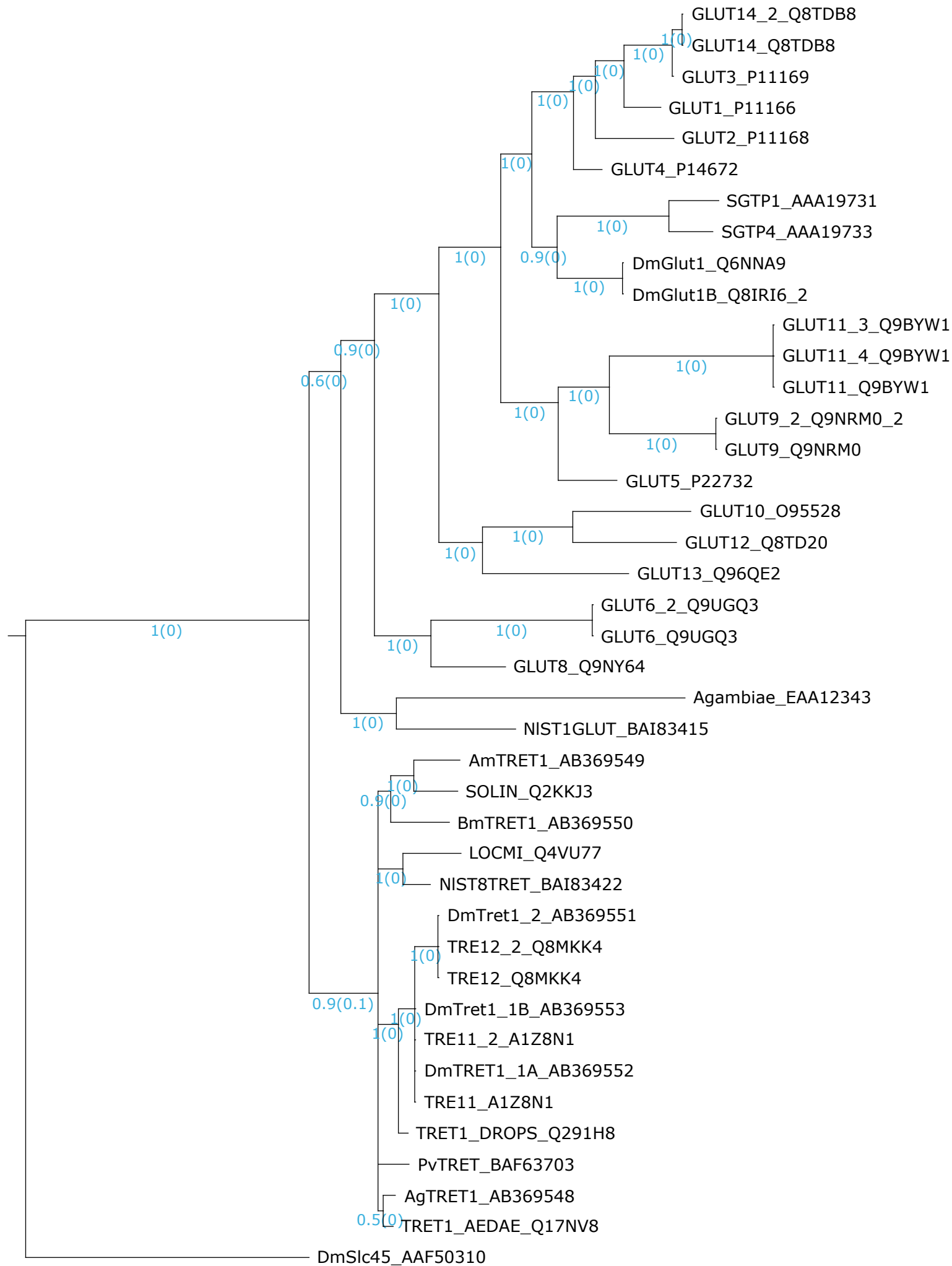

Supplement: Additional file 7: Figure S2. — MrBayes phylogenetic tree of trehalose and glucose transporters family. Maximum likelihood (ML) phylogenetic analysis for trehalose and glucose transporters. Bootstraps values are shown. (PDF 17 kb) [file 13071_2015_1079_MOESM7_ESM.pdf]

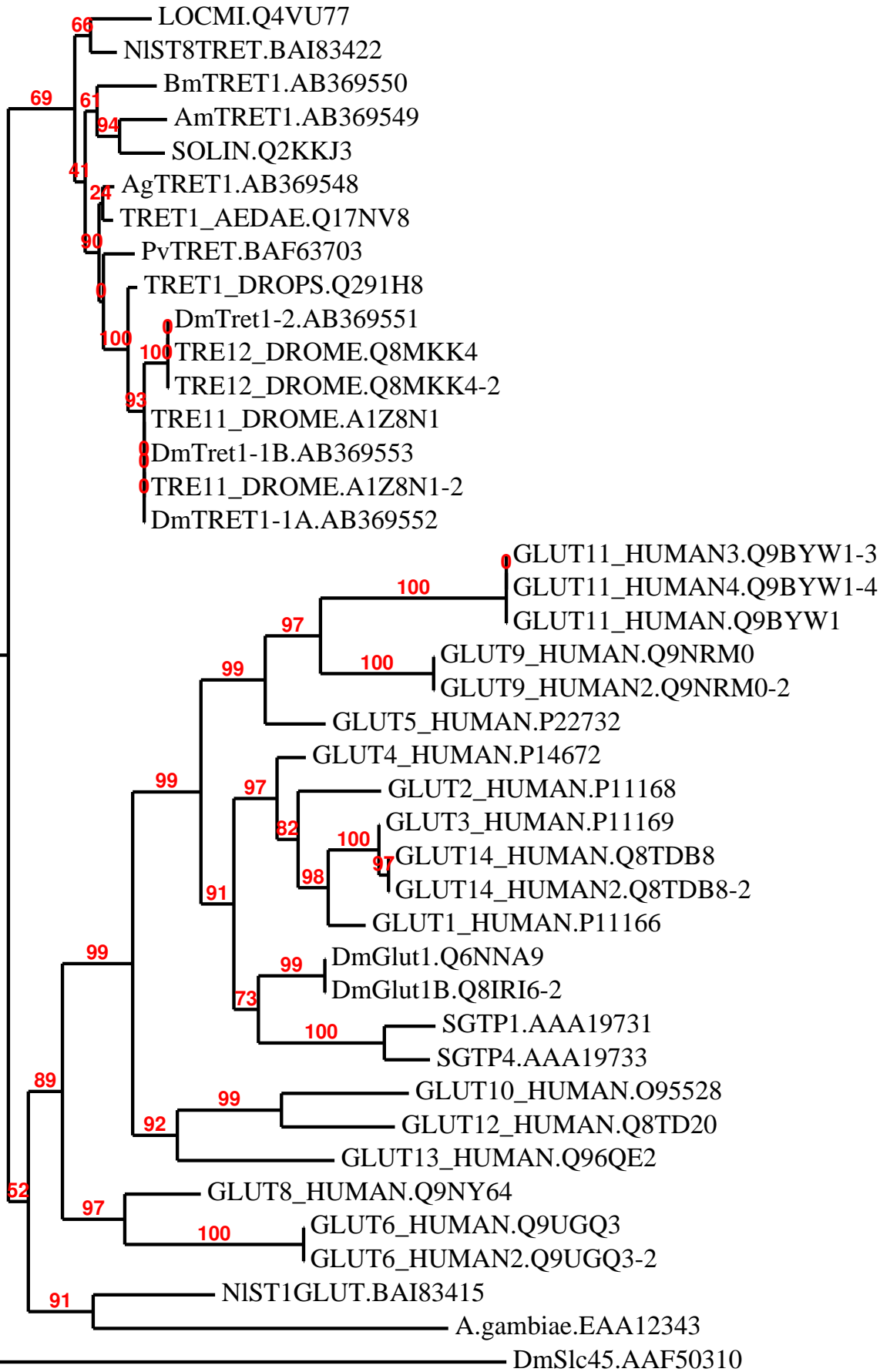

Supplement: Additional file 8: Figure S3. — Maximum likelihood phylogenetic tree of trehalose and glucose transporters family. Maximum likelihood (ML) phylogenetic analysis for trehalose and glucose transporters. Bootstraps values are shown. (PDF 5 kb) [file 13071_2015_1079_MOESM8_ESM.pdf]
