# Supplementary material for: Gene expression changes in the salivary glands of Anopheles coluzzii elicited by Plasmodium berghei infection
Source: Parasit Vectors. 2015 Sep 23;8:485. doi: 10.1186/s13071-015-1079-8 (PMC4580310; doi:10.1186/s13071-015-1079-8)
Supplement: Additional file 9: — File S1. Alignment for trehalose and glucose transporters used in the phylogenetic analysis. (DOC 113 kb) [file 13071_2015_1079_MOESM9_ESM.doc]

A.gambiae.EAA12 ------------------------------------------------------------

AgTRET1.AB36954 ------------------------------------------------------------

AgGLUT.EAA11842 ------------------------------------------------------------

TRET1_AEDAE.Q17 MFGNEMDDTR---------DPL----------------QYGYQRVNTGEGSLSTSTTGTS

PvTRET.BAF63703 ------------------------------------------------------------

DmTRET1-1A.AB36 ------------------------------------------------------------

TRE11_DROME.A1Z MSGR---DNRGAGGGGGGHQPLSNAMGKLKEKLTRVGDELGYHRV---ESNLSTSNTATS

DmTret1-1B.AB36 M-----------------------------------------------------------

TRE11_DROME.A1Z M-----------------------------------------------------------

TRET1_DROPS.Q29 MSGR---DNRGAGGGGGGHQPLSSAMGKLKEKLTRAGDDQGYHRV---ESNLSTSNTATS

DmTret1-2.AB369 M-----------------------------------------------------------

TRE12_DROME.Q8M M-----------------------------------------------------------

TRE12_DROME.Q8M ------------------------------------------------------------

NlST8TRET.BAI83 ------------------------------------------------------------

AmTRET1.AB36954 ------------------------------------------------------------

SOLIN.Q2KKJ3 ------------------------------------------------------------

LOCMI.Q4VU77 -----------AGSG---------------------------------------------

BmTRET1.AB36955 ------------------------------------------------------------

NlST1GLUT.BAI83 ------------------------------------------------------------

GLUT6_HUMAN.Q9U ------------------------------------------------------------

GLUT6_HUMAN2.Q9 ------------------------------------------------------------

GLUT8_HUMAN.Q9N ------------------------------------------------------------

GLUT13_HUMAN.Q9 MS-------RKASEN---------------------------------------------

GLUT1_HUMAN.P11 ------------------------------------------------------------

GLUT4_HUMAN.P14 ------------------------------------------------------------

GLUT3_HUMAN.P11 ------------------------------------------------------------

GLUT14_HUMAN.Q8 ------------------------------------------------------------

GLUT14_HUMAN2.Q ------------------------------------------------------------

GLUT2_HUMAN.P11 ------------------------------------------------------------

DmGlut1.Q6NNA9 ------------------------------------------------------------

DmGlut1B.Q8IRI6 ------------------------------------------------------------

GLUT5_HUMAN.P22 ------------------------------------------------------------

GLUT9_HUMAN.Q9N ------------------------------------------------------------

GLUT9_HUMAN2.Q9 ------------------------------------------------------------

GLUT11_HUMAN.Q9 ------------------------------------------------------------

GLUT11_HUMAN3.Q ------------------------------------------------------------

GLUT11_HUMAN4.Q ------------------------------------------------------------

SGTP1.AAA19731 ------------------------------------------------------------

SGTP4.AAA19733 ------------------------------------------------------------

GLUT10_HUMAN.O9 ------------------------------------------------------------

GLUT12_HUMAN.Q8 ------------------------------------------------------------

DmSlc45.AAF5031 ------------------------------------------------------------

A.gambiae.EAA12 ------------------------------------------------------------

AgTRET1.AB36954 ------------------------------------------------------------

AgGLUT.EAA11842 ------------------------------------------------------------

TRET1_AEDAE.Q17 LDTIVLDTNAEDLNSTPRVGAQR--------------------------TFSPILETDDT

PvTRET.BAF63703 ------------------------------------------------------------

DmTRET1-1A.AB36 ------------------------------------------------------------

TRE11_DROME.A1Z LDTIL----PEDPFLFPQVSPQRHP-------QNTVRTQ-RLLEDEPPLSFRPLLEDDDI

DmTret1-1B.AB36 ------------------------------------------------------------

TRE11_DROME.A1Z ------------------------------------------------------------

TRET1_DROPS.Q29 LDTIL----PEDPFLFPQAAPQRHPLPRPQQQQQQQRQQLRLLEDEPPLSFRPLLEDDDI

DmTret1-2.AB369 ------------------------------------------------------------

TRE12_DROME.Q8M ------------------------------------------------------------

TRE12_DROME.Q8M ------------------------------------------------------------

NlST8TRET.BAI83 ------------------------------------------------------------

AmTRET1.AB36954 ------------------------------------------------------------

SOLIN.Q2KKJ3 ------------------------------------------------------------

LOCMI.Q4VU77 ------------------------------------------------------------

BmTRET1.AB36955 ------------------------------------------------------------

NlST1GLUT.BAI83 ------------------------------------------------------------

GLUT6_HUMAN.Q9U ------------------------------------------------------------

GLUT6_HUMAN2.Q9 ------------------------------------------------------------

GLUT8_HUMAN.Q9N ------------------------------------------------------------

GLUT13_HUMAN.Q9 ------------------------------------------------------------

GLUT1_HUMAN.P11 ------------------------------------------------------------

GLUT4_HUMAN.P14 ------------------------------------------------------------

GLUT3_HUMAN.P11 ------------------------------------------------------------

GLUT14_HUMAN.Q8 ------------------------------------------------------------

GLUT14_HUMAN2.Q ------------------------------------------------------------

GLUT2_HUMAN.P11 ------------------------------------------------------------

DmGlut1.Q6NNA9 ------------------------------------------------------------

DmGlut1B.Q8IRI6 ------------------------------------------------------------

GLUT5_HUMAN.P22 ------------------------------------------------------------

GLUT9_HUMAN.Q9N ------------------------------------------------------------

GLUT9_HUMAN2.Q9 ------------------------------------------------------------

GLUT11_HUMAN.Q9 ------------------------------------------------------------

GLUT11_HUMAN3.Q ------------------------------------------------------------

GLUT11_HUMAN4.Q ------------------------------------------------------------

SGTP1.AAA19731 ------------------------------------------------------------

SGTP4.AAA19733 ------------------------------------------------------------

GLUT10_HUMAN.O9 ------------------------------------------------------------

GLUT12_HUMAN.Q8 ------------------------------------------------------------

DmSlc45.AAF5031 ------------------------------------------------------------

A.gambiae.EAA12 ------------------------------------------------------------

AgTRET1.AB36954 ------------------------------------------------------------

AgGLUT.EAA11842 ------------------------------------------------------------

TRET1_AEDAE.Q17 N-----PFLD------------------PPPPG-----------QKSPPAVGEAKAKSKS

PvTRET.BAF63703 ------------------------------------------------------------

DmTRET1-1A.AB36 ------------------------------------------------------------

TRE11_DROME.A1Z NEPPT----QQQQRTPLRASGSLELTPLPPPPTSLEIREHRDRQQRG--AQGDELQRSKQ

DmTret1-1B.AB36 ------------------------------------------------------------

TRE11_DROME.A1Z ------------------------------------------------------------

TRET1_DROPS.Q29 NEPPTQPFQQQQQRTPLRASGSLELTPLPPPPTSQEIREHRDRQQRSVPVPVEDLQRSKQ

DmTret1-2.AB369 ------------------------------------------------------------

TRE12_DROME.Q8M ------------------------------------------------------------

TRE12_DROME.Q8M ------------------------------------------------------------

NlST8TRET.BAI83 ------------------------------------------------------------

AmTRET1.AB36954 ------------------------------------------------------------

SOLIN.Q2KKJ3 ------------------------------------------------------------

LOCMI.Q4VU77 ------------------------------------------------------------

BmTRET1.AB36955 ------------------------------------------------------------

NlST1GLUT.BAI83 ------------------------------------------------------------

GLUT6_HUMAN.Q9U ------------------------------------------------------------

GLUT6_HUMAN2.Q9 ------------------------------------------------------------

GLUT8_HUMAN.Q9N ------------------------------------------------------------

GLUT13_HUMAN.Q9 ------------------------------------------------------------

GLUT1_HUMAN.P11 ------------------------------------------------------------

GLUT4_HUMAN.P14 ------------------------------------------------------------

GLUT3_HUMAN.P11 ------------------------------------------------------------

GLUT14_HUMAN.Q8 ------------------------------------------------------------

GLUT14_HUMAN2.Q ------------------------------------------------------------

GLUT2_HUMAN.P11 ------------------------------------------------------------

DmGlut1.Q6NNA9 ------------------------------------------------------------

DmGlut1B.Q8IRI6 ------------------------------------------------------------

GLUT5_HUMAN.P22 ------------------------------------------------------------

GLUT9_HUMAN.Q9N ------------------------------------------------------------

GLUT9_HUMAN2.Q9 ------------------------------------------------------------

GLUT11_HUMAN.Q9 ------------------------------------------------------------

GLUT11_HUMAN3.Q ------------------------------------------------------------

GLUT11_HUMAN4.Q ------------------------------------------------------------

SGTP1.AAA19731 ------------------------------------------------------------

SGTP4.AAA19733 ------------------------------------------------------------

GLUT10_HUMAN.O9 ------------------------------------------------------------

GLUT12_HUMAN.Q8 ------------------------------------------------------------

DmSlc45.AAF5031 ------------------------------------------------------------

A.gambiae.EAA12 ------------------------------------------------------------

AgTRET1.AB36954 ------------------------------------------------------------

AgGLUT.EAA11842 ------------------------------------------------------------

TRET1_AEDAE.Q17 SLKGSRVSFDQED----------RFDETDE-GFRKQREHFQKHKSHSTSEHKNQLIKELR

PvTRET.BAF63703 ------------------------------------------------------------

DmTRET1-1A.AB36 ------------------------------------------------------------

TRE11_DROME.A1Z SLKGSRVSFERRDTGNSNTNSNKAAESSDEDSFEEKRTGFQQQKATSV-DHKG-ILKDLK

DmTret1-1B.AB36 ------------------------------------------------------------

TRE11_DROME.A1Z ------------------------------------------------------------

TRET1_DROPS.Q29 SLKGSRVSFEK---NNASSKPPAQAESSDEDSFEDKRIGFQQQKATSV-DHKG-ILKDLK

DmTret1-2.AB369 ------------------------------------------------------------

TRE12_DROME.Q8M ------------------------------------------------------------

TRE12_DROME.Q8M ------------------------------------------------------------

NlST8TRET.BAI83 ------------------------------------------------------------

AmTRET1.AB36954 ------------------------------------------------------------

SOLIN.Q2KKJ3 ------------------------------------------------------------

LOCMI.Q4VU77 ------------------------------------------------------------

BmTRET1.AB36955 ------------------------------------------------------------

NlST1GLUT.BAI83 ------------------------------------------------------------

GLUT6_HUMAN.Q9U ------------------------------------------------------------

GLUT6_HUMAN2.Q9 ------------------------------------------------------------

GLUT8_HUMAN.Q9N ------------------------------------------------------------

GLUT13_HUMAN.Q9 ------------------------------------------------------------

GLUT1_HUMAN.P11 ------------------------------------------------------------

GLUT4_HUMAN.P14 ------------------------------------------------------------

GLUT3_HUMAN.P11 ------------------------------------------------------------

GLUT14_HUMAN.Q8 ------------------------------------------------------------

GLUT14_HUMAN2.Q ------------------------------------------------------------

GLUT2_HUMAN.P11 ------------------------------------------------------------

DmGlut1.Q6NNA9 ------------------------------------------------------------

DmGlut1B.Q8IRI6 ------------------------------------------------------------

GLUT5_HUMAN.P22 ------------------------------------------------------------

GLUT9_HUMAN.Q9N ------------------------------------------------------------

GLUT9_HUMAN2.Q9 ------------------------------------------------------------

GLUT11_HUMAN.Q9 ------------------------------------------------------------

GLUT11_HUMAN3.Q ------------------------------------------------------------

GLUT11_HUMAN4.Q ------------------------------------------------------------

SGTP1.AAA19731 ------------------------------------------------------------

SGTP4.AAA19733 ------------------------------------------------------------

GLUT10_HUMAN.O9 ------------------------------------------------------------

GLUT12_HUMAN.Q8 ------------------------------------------------------------

DmSlc45.AAF5031 ------------------------------------------------------------

A.gambiae.EAA12 ------------------------------------------------------------

AgTRET1.AB36954 ------------------------------------------------------------

AgGLUT.EAA11842 ------------------------------------------------------------

TRET1_AEDAE.Q17 HLLAADNRRQFQGKKHVSLDVQSSKVLEELLKASSSEDDFEGQRKQFQERKHKSLDARHI

PvTRET.BAF63703 ------------------------------------------------------------

DmTRET1-1A.AB36 ------------------------------------------------------------

TRE11_DROME.A1Z HILANDNRRQFQAKKHVSLDVKGTRFLQDLLKESSSEEEFHKTRREFQGRKHQSLDPR-V

DmTret1-1B.AB36 ------------------------------------------------------------

TRE11_DROME.A1Z ------------------------------------------------------------

TRET1_DROPS.Q29 HILANDNRRQFQAKKHVSLDVKGTRFLQDLLKESSSEEEFHKTRREFQGRKHQSLDPR-V

DmTret1-2.AB369 ------------------------------------------------------------

TRE12_DROME.Q8M ------------------------------------------------------------

TRE12_DROME.Q8M ------------------------------------------------------------

NlST8TRET.BAI83 ------------------------------------------------------------

AmTRET1.AB36954 ------------------------------------------------------------

SOLIN.Q2KKJ3 ------------------------------------------------------------

LOCMI.Q4VU77 ------------------------------------------------------------

BmTRET1.AB36955 ------------------------------------------------------------

NlST1GLUT.BAI83 ------------------------------------------------------------

GLUT6_HUMAN.Q9U ------------------------------------------------------------

GLUT6_HUMAN2.Q9 ------------------------------------------------------------

GLUT8_HUMAN.Q9N ------------------------------------------------------------

GLUT13_HUMAN.Q9 -----------------------------------------------------------V

GLUT1_HUMAN.P11 ------------------------------------------------------------

GLUT4_HUMAN.P14 ------------------------------------------------------------

GLUT3_HUMAN.P11 ------------------------------------------------------------

GLUT14_HUMAN.Q8 ------------------------------------------------------------

GLUT14_HUMAN2.Q ------------------------------------------------------------

GLUT2_HUMAN.P11 ------------------------------------------------------------

DmGlut1.Q6NNA9 ------------------------------------------------------------

DmGlut1B.Q8IRI6 ------------------------------------------------------------

GLUT5_HUMAN.P22 ------------------------------------------------------------

GLUT9_HUMAN.Q9N ------------------------------------------------------------

GLUT9_HUMAN2.Q9 ------------------------------------------------------------

GLUT11_HUMAN.Q9 ------------------------------------------------------------

GLUT11_HUMAN3.Q ------------------------------------------------------------

GLUT11_HUMAN4.Q ------------------------------------------------------------

SGTP1.AAA19731 ------------------------------------------------------------

SGTP4.AAA19733 ------------------------------------------------------------

GLUT10_HUMAN.O9 ------------------------------------------------------------

GLUT12_HUMAN.Q8 ------------------------------------------------------------

DmSlc45.AAF5031 ------------------------------------------------------------

A.gambiae.EAA12 ------------------------------------------------------------

AgTRET1.AB36954 ------------------------------------------------------------

AgGLUT.EAA11842 ------------------------------------------------------------

TRET1_AEDAE.Q17 SFKFEKEPTPSSSEEDFE----PSTSLLKIDADITKPVIIDLKVNRAQSDQALTQFQKNL

PvTRET.BAF63703 ------------------------------------------------------------

DmTRET1-1A.AB36 ------------------------------------------------------------

TRE11_DROME.A1Z TFKLDKVLQGSSTDSDEEGEDAEHKRLIHRPKDITKPVIIDLK-----------------

DmTret1-1B.AB36 -----KIL----------------------------------------------------

TRE11_DROME.A1Z -----KIL----------------------------------------------------

TRET1_DROPS.Q29 TFKLDKVLQGSSTDSDEEGDDPEHKRLIHRPKDITKPLIIDLK-----------------

DmTret1-2.AB369 -----KIL----------------------------------------------------

TRE12_DROME.Q8M -----KIL----------------------------------------------------

TRE12_DROME.Q8M ------------------------------------------------------------

NlST8TRET.BAI83 ------------------------------------------------------------

AmTRET1.AB36954 ------------------------------------------------------------

SOLIN.Q2KKJ3 ------------------------------------------------------------

LOCMI.Q4VU77 ------------------------------------------------------------

BmTRET1.AB36955 ------------------------------------------------------------

NlST1GLUT.BAI83 ------------------------------------------------------------

GLUT6_HUMAN.Q9U ------------------------------------------------------------

GLUT6_HUMAN2.Q9 ------------------------------------------------------------

GLUT8_HUMAN.Q9N ------------------------------------------------------------

GLUT13_HUMAN.Q9 EYTL--------------------------------------------------------

GLUT1_HUMAN.P11 ------------------------------------------------------------

GLUT4_HUMAN.P14 ------------------------------------------------------------

GLUT3_HUMAN.P11 ------------------------------------------------------------

GLUT14_HUMAN.Q8 ------------------------------------------------------------

GLUT14_HUMAN2.Q ------------------------------------------------------------

GLUT2_HUMAN.P11 ------------------------------------------------------------

DmGlut1.Q6NNA9 ------------------------------------------------------------

DmGlut1B.Q8IRI6 ------------------------------------------------------------

GLUT5_HUMAN.P22 ------------------------------------------------------------

GLUT9_HUMAN.Q9N ------------------------------------------------------------

GLUT9_HUMAN2.Q9 ------------------------------------------------------------

GLUT11_HUMAN.Q9 ------------------------------------------------------------

GLUT11_HUMAN3.Q ------------------------------------------------------------

GLUT11_HUMAN4.Q ------------------------------------------------------------

SGTP1.AAA19731 ------------------------------------------------------------

SGTP4.AAA19733 ------------------------------------------------------------

GLUT10_HUMAN.O9 ------------------------------------------------------------

GLUT12_HUMAN.Q8 ------------------------------------------------------------

DmSlc45.AAF5031 ------------------------------------------------------------

A.gambiae.EAA12 ------------------------------------------MAST--------------

AgTRET1.AB36954 ------------------------------------------MEM--GTKEE--------

AgGLUT.EAA11842 ------------------------------------------VKIL--------------

TRET1_AEDAE.Q17 AHFQDLESSEDEDYISSRKHFQQAKSMSTDSRKSNKSIRFFEMEM--GTKEE--------

PvTRET.BAF63703 ------------------------------------------MEL--NNKED--------

DmTRET1-1A.AB36 ------------------------------------------MDEMDNKRGE--------

TRE11_DROME.A1Z ----DLESESDEDFLTSRQHFQQQRSISTDSRKSR---RLYEMDEMDNKRGE--------

DmTret1-1B.AB36 ------------------------------------------------------------

TRE11_DROME.A1Z ------------------------------------------------------------

TRET1_DROPS.Q29 ----DLESESDEDFHTSRQHFQQQRSISTDSRKSR---RPYEMDEMGNKRGE--------

DmTret1-2.AB369 ------------------------------------------------------------

TRE12_DROME.Q8M ------------------------------------------------------------

TRE12_DROME.Q8M ------------------------------------------------------------

NlST8TRET.BAI83 ------------------------------------------MKRL--------------

AmTRET1.AB36954 ------------------------------------------MGVE-NTKQT--------

SOLIN.Q2KKJ3 ------------------------------------------------------------

LOCMI.Q4VU77 ------------------------------------------LSGL--------------

BmTRET1.AB36955 ------------------------------------------MEME--IKDE--------

NlST1GLUT.BAI83 ------------------------------------------MS----------------

GLUT6_HUMAN.Q9U ------------------------------------------MQ-------EPL------

GLUT6_HUMAN2.Q9 ------------------------------------------MQ-------EPL------

GLUT8_HUMAN.Q9N ------------------------------------------MTPEDPEETQPL------

GLUT13_HUMAN.Q9 ------------------------RSLSS------------LMGERRRKQPEPD------

GLUT1_HUMAN.P11 ------------------------------------------ME----------------

GLUT4_HUMAN.P14 ------------------------------------------MP----------------

GLUT3_HUMAN.P11 ------------------------------------------MG----------------

GLUT14_HUMAN.Q8 ------------------------------------------MEFHN-------------

GLUT14_HUMAN2.Q ------------------------------------------MD----------------

GLUT2_HUMAN.P11 ------------------------------------------MT----------------

DmGlut1.Q6NNA9 ------------------------------------------MAF---------------

DmGlut1B.Q8IRI6 ------------------------------------------MAF---------------

GLUT5_HUMAN.P22 ------------------------------------------ME----------------

GLUT9_HUMAN.Q9N ------------------------------------------MA-RKQNRNSKELGLVPL

GLUT9_HUMAN2.Q9 ------------------------------------------MKLSKKDRGEDE------

GLUT11_HUMAN.Q9 ------------------------------------------MR----------------

GLUT11_HUMAN3.Q ------------------------------------------ML----------------

GLUT11_HUMAN4.Q ------------------------------------------ME----------------

SGTP1.AAA19731 ------------------------------------------MG----------------

SGTP4.AAA19733 ------------------------------------------MG----------------

GLUT10_HUMAN.O9 ------------------------------------------MG----------------

GLUT12_HUMAN.Q8 ------------------------------------------MVPVENTEGPSL------

DmSlc45.AAF5031 ------------------------------------------MV----------------

A.gambiae.EAA12 ------------------------------------------------------------

AgTRET1.AB36954 ----------NMRTAVPFVRQITEEG--------KPKLEV------YRPTTNPIY-----

AgGLUT.EAA11842 ---------------------MRADT--------HVSFSV--------PIEEPVA-----

TRET1_AEDAE.Q17 ----------NIRTAVPFVRQITEDG--------KPKLEV------YRPTTNPIF-----

PvTRET.BAF63703 ----------SPRHTVPFVRQITEDG--------KAKLEI------YRPTTNPIY-----

DmTRET1-1A.AB36 ----------NIRHAVPFVRQITEDG--------KPKLEV------YRPTTNPIY-----

TRE11_DROME.A1Z ----------NIRHAVPFVRQITEDG--------KPKLEV------YRPTTNPIY-----

DmTret1-1B.AB36 ---------------------MRADT--------HVSFSV------PVEEPKAIC-----

TRE11_DROME.A1Z ---------------------MRADT--------HVSFSV------PVEEPKAIC-----

TRET1_DROPS.Q29 ----------NIRHAVPFVRQITEDG--------KPKLEV------YRPTTNPIY-----

DmTret1-2.AB369 ---------------------MRADT--------HVSFSV------PADGLKANF-----

TRE12_DROME.Q8M ---------------------MRADT--------HVSFSV------PADGLKANF-----

TRE12_DROME.Q8M ------------------------------------------------------------

NlST8TRET.BAI83 ---------------------LRADT--------HPSIHVP-----SRGSAEVNF-----

AmTRET1.AB36954 --------------MSSQNIKPAKDS--------DDVLHTQF----KEVKRSPMR-----

SOLIN.Q2KKJ3 --------------MDPQDVKLPKDL--------QEPIRLPSSEFQREPKSSWIR-----

LOCMI.Q4VU77 --------------VDSKQIDTVEDG--------DVPARV------GRGR----------

BmTRET1.AB36955 ----------NLRNSVPFVRQLSTDSVKTK-TEYDNEDGTP-----YKSTTQKLF-----

NlST1GLUT.BAI83 ----------------TKATTVSAQT--------------------LVSTAVPAA-----

GLUT6_HUMAN.Q9U ---------------------LGAEG-----PDYDTFPEKPPPSPGDRARVGTLQ-----

GLUT6_HUMAN2.Q9 ---------------------LGAEG-----PDYDTFPEKPPPSPGDRARVGTLQ-----

GLUT8_HUMAN.Q9N ---LGPPGG-----SAPR------------------------------------------

GLUT13_HUMAN.Q9 A--ASAAGECSLLAAAESSTSLQSAGAGGGGVG----------DLERAARRQFQQD----

GLUT1_HUMAN.P11 -------------------------------------------------PSSKKL-----

GLUT4_HUMAN.P14 ---------------SGFQQIGSED----------------------GEPPQQRV-----

GLUT3_HUMAN.P11 ---------------------------------------------------TQKV-----

GLUT14_HUMAN.Q8 -------GG-HVSGIGGFLVSLTSR----------------------MKPHTLAV-----

GLUT14_HUMAN2.Q --------------------------------------------------NRQNV-----

GLUT2_HUMAN.P11 ---------------------------------------------------EDKV-----

DmGlut1.Q6NNA9 -------------------------------------------------LCAPGL-----

DmGlut1B.Q8IRI6 -------------------------------------------------LCAPGL-----

GLUT5_HUMAN.P22 ----------------------QQD----------------------QSMKEGRL-----

GLUT9_HUMAN.Q9N TDDTSHAGP-----PGPGRALLECDHLRSGV---------------PGGRRRKDW-----

GLUT9_HUMAN2.Q9 ----------------------ESD----------------------SAKKKLDW-----

GLUT11_HUMAN.Q9 ---------------------------------------------ALRRLIQGR------

GLUT11_HUMAN3.Q ----------------------HAL-LR-------------------SRMIQGR------

GLUT11_HUMAN4.Q -------------------DELEPS-LR------------------PRTQIQGR------

SGTP1.AAA19731 ------------------------------------------------VASNNGI-----

SGTP4.AAA19733 --------------------------------------------------SGKKF-----

GLUT10_HUMAN.O9 ------------------------------------------------------------

GLUT12_HUMAN.Q8 --------------LNQKGTAVETEG--------------------SGSRHPPWARGC--

DmSlc45.AAF5031 ----------GVTADASQANQLSSVR--------NPMIKYMLKTRENHAREQDRD-YSHV

A.gambiae.EAA12 --RGVRNQIFAT-GVMNLINLSHGAALGWVSPYLPILMS---------------------

AgTRET1.AB36954 ----IWTQVLAA-LSVSLGSMVVGFSSAYTSPALVSMKD---------------------

AgGLUT.EAA11842 --KCTFSQVLAA-LSVSLGSMVVGFSSAYTSPALVSMKD---------------------

TRET1_AEDAE.Q17 ----IWTQVLAA-LSVSLGSMVVGFSSAYTSPALVSMKD---------------------

PvTRET.BAF63703 ----IYTQILAA-IAVSMGSMVVGFASAYTSPALVSMQN---------------------

DmTRET1-1A.AB36 ----IWTQVLAA-LSVSLGSLVVGFVSAYTSPALVSMTD---------------------

TRE11_DROME.A1Z ----IWTQVLAA-LSVSLGSLVVGFVSAYTSPALVSMTD---------------------

DmTret1-1B.AB36 ----TFSQVLAA-LSVSLGSLVVGFVSAYTSPALVSMTD---------------------

TRE11_DROME.A1Z ----TFSQVLAA-LSVSLGSLVVGFVSAYTSPALVSMTD---------------------

TRET1_DROPS.Q29 ----IWTQVLAA-LSVSLGSLVVGFVSAYTSPALVSMTN---------------------

DmTret1-2.AB369 ----TFSQVLAA-LSVSLCSLVVGFVSAYTSPALVSMTD---------------------

TRE12_DROME.Q8M ----TFSQVLAA-LSVSLCSLVVGFVSAYTSPALVSMTD---------------------

TRE12_DROME.Q8M ------------------------------------MTD---------------------

NlST8TRET.BAI83 ----TWSQVLAA-VSVSLGSMVVGFSSAYTSPAIASMNS---------------------

AmTRET1.AB36954 ----YTMQLLAA-LAVSMASLMIGYSSSYTSPALVSMRD---------------------

SOLIN.Q2KKJ3 ----YAAQILAA-LSVSLGSMQVGYSSSYTSPALVSMRD---------------------

LOCMI.Q4VU77 ----LWTQVLAS-VSVSTGSLVVGFSSAYTSPALASMKA---------------------

BmTRET1.AB36955 ----LWTQLLAA-FAVSVGSMNVGFSSGYTSPAVLTMN----------------------

NlST1GLUT.BAI83 ----KLPQYVAA-LIATIGGFCLGTVLGWTSPVLTSLS----------------------

GLUT6_HUMAN.Q9U ----NKRVFLAT-FAAVLGNFSFGYALVYTSPVIPALER---------------------

GLUT6_HUMAN2.Q9 ----NKRVFLAT-FAAVLGNFSFGYALVYTSPVIPALER---------------------

GLUT8_HUMAN.Q9N ----GRRVFLAA-FAAALGPLSFGFALGYSSPAIPSLQR---------------------

GLUT13_HUMAN.Q9 --ETPAFVYVVA-VFSALGGFLFGYDTGVVSGAMLLLK----------------------

GLUT1_HUMAN.P11 ----TGRLMLAV-GGAVLGSLQFGYNTGVINAPQKVIEEFY--------------NQTWV

GLUT4_HUMAN.P14 ----TGTLVLAV-FSAVLGSLQFGYNIGVINAPQKVIEQSY--------------NETWL

GLUT3_HUMAN.P11 ----TPALIFAI-TVATIGSFQFGYNTGVINAPEKIIKEFI--------------NKTLT

GLUT14_HUMAN.Q8 ----TPALIFAI-TVATIGSFQFGYNTGVINAPETIIKEFI--------------NKTLT

GLUT14_HUMAN2.Q ----TPALIFAI-TVATIGSFQFGYNTGVINAPETIIKEFI--------------NKTLT

GLUT2_HUMAN.P11 ----TGTLVFTV-ITAVLGSFQFGYDIGVINAPQQVIISHYRHVLGVPLDDRKAINNYVI

DmGlut1.Q6NNA9 ----TFFLTYSI-FSAVLGMLQFGYNTGVINAPEKNIENFM--------------KDVYK

DmGlut1B.Q8IRI6 ----TFFLTYSI-FSAVLGMLQFGYNTGVINAPEKNIENFM--------------KDVYK

GLUT5_HUMAN.P22 ----TLVLALATLIAAFGSSFQYGYNVAAVNSPALLMQQFY--------------NETYY

GLUT9_HUMAN.Q9N ----SCSLLVASLAGAFGSSFLYGYNLSVVNAPTPYIKAFY--------------NESWE

GLUT9_HUMAN2.Q9 ----SCSLLVASLAGAFGSSFLYGYNLSVVNAPTPYIKAFY--------------NESWE

GLUT11_HUMAN.Q9 ------ILLLTICAAGIGGTFQFGYNLSIINAPTLHIQEFT--------------NETWQ

GLUT11_HUMAN3.Q ------ILLLTICAAGIGGTFQFGYNLSIINAPTLHIQEFT--------------NETWQ

GLUT11_HUMAN4.Q ------ILLLTICAAGIGGTFQFGYNLSIINAPTLHIQEFT--------------NETWQ

SGTP1.AAA19731 ----TGKLVLTVLITCVGSSFLIGYNLGVLNLPRRNIEIYF--------------NETVV

SGTP4.AAA19733 ----TKSLSLSVLLACLGSSFTIGYNLGVLNLPGENIKEFL--------------SRTML

GLUT10_HUMAN.O9 --HSPPVLPLCA-SVSLLGGLTFGYELAVISGALLPLQ----------------------

GLUT12_HUMAN.Q8 ----GMFTFLSS-VTAAVSGLLVGYELGIISGALLQIK----------------------

DmSlc45.AAF5031 FRRKTRFEMFRLSAIAMAIEFAYAAETSFVSPILLQIGVDHKH-----------MSMTW-

:

A.gambiae.EAA12 --------------------PDQDLLSTGPVTVEQGSW-------------IGSILCLGA

AgTRET1.AB36954 -----------------------RNITSFEVTDQSGSW-------------VGGIMPLAG

AgGLUT.EAA11842 -----------------------RNITSFEVTDQSGSW-------------VGGIMPLAG

TRET1_AEDAE.Q17 -----------------------RNITSFEVTDQSGSW-------------VGGIMPLAG

PvTRET.BAF63703 -----------------------TTITSFKVTEQEASW-------------VGGIMPLAG

DmTRET1-1A.AB36 -----------------------RNITSFEVTQDAGSW-------------VGGIMPLAG

TRE11_DROME.A1Z -----------------------RNITSFEVTQDAGSW-------------VGGIMPLAG

DmTret1-1B.AB36 -----------------------RNITSFEVTQDAGSW-------------VGGIMPLAG

TRE11_DROME.A1Z -----------------------RNITSFEVTQDAGSW-------------VGGIMPLAG

TRET1_DROPS.Q29 -----------------------RNMTSFEVTPQAASW-------------VGGIMPLAG

DmTret1-2.AB369 -----------------------RTITSFEVTKDAGSW-------------VGGIMPLAA

TRE12_DROME.Q8M -----------------------RTITSFEVTKDAGSW-------------VGGIMPLAA

TRE12_DROME.Q8M -----------------------RTITSFEVTKDAGSW-------------VGGIMPLAA

NlST8TRET.BAI83 ------------------------NASSLHVTPQEESW-------------IGSLMPLCA

AmTRET1.AB36954 -----------------------NTTATFEVTMDMAMW-------------IGSIMPLSA

SOLIN.Q2KKJ3 -----------------------DATATFEVTKHMSMW-------------IGSLMPLSA

LOCMI.Q4VU77 -----------------------DTNSTITVDEQQESW-------------IGSLMPLAA

BmTRET1.AB36955 --------------------------ITLDITKEEITW-------------VGGLMPLAA

NlST1GLUT.BAI83 ------------------------DYYGFEVNVDSQAW-------------IGSIMAIGA

GLUT6_HUMAN.Q9U -----------------------SLDPDLHLTKSQASW-------------FGSVFTLGA

GLUT6_HUMAN2.Q9 -----------------------SLDPDLHLTKSQASW-------------FGSVFTLGA

GLUT8_HUMAN.Q9N -----------------------AAPPAPRLDDAAASW-------------FGAVVTLGA

GLUT13_HUMAN.Q9 --------------------------RQLSLDALWQEL-------------LVSSTVGAA

GLUT1_HUMAN.P11 HRYG--------------------ESILPTTLTTLWSL-------------SVAIFSVGG

GLUT4_HUMAN.P14 GRQG----------------PEGPSSIPPGTLTTLWAL-------------SVAIFSVGG

GLUT3_HUMAN.P11 DKGN--------------------APPSEVLLTSLWSL-------------SVAIFSVGG

GLUT14_HUMAN.Q8 DKAN--------------------APPSEVLLTNLWSL-------------SVAIFSVGG

GLUT14_HUMAN2.Q DKAN--------------------APPSEVLLTNLWSL-------------SVAIFSVGG

GLUT2_HUMAN.P11 NSTDELPTISYSMNPKPTPWAEEETVAAAQLITMLWSL-------------SVSSFAVGG

DmGlut1.Q6NNA9 DRYG--------------------EDISEEFIQQLYSV-------------AVSIFAIGG

DmGlut1B.Q8IRI6 DRYG--------------------EDISEEFIQQLYSV-------------AVSIFAIGG

GLUT5_HUMAN.P22 GRTG--------------------EFMEDFPLTLLWSV-------------TVSMFPFGG

GLUT9_HUMAN.Q9N RRHG--------------------RPIDPDTLTLLWSV-------------TVSIFAIGG

GLUT9_HUMAN2.Q9 RRHG--------------------RPIDPDTLTLLWSV-------------TVSIFAIGG

GLUT11_HUMAN.Q9 ARTG--------------------EPLPDHLVLLMWSL-------------IVSLYPLGG

GLUT11_HUMAN3.Q ARTG--------------------EPLPDHLVLLMWSL-------------IVSLYPLGG

GLUT11_HUMAN4.Q ARTG--------------------EPLPDHLVLLMWSL-------------IVSLYPLGG

SGTP1.AAA19731 PNTPEL------------------------DSSFFYTH-------------VSTIFVVAA

SGTP4.AAA19733 GKNA----------------SEAENTANLVTPSFLYAQ-------------VSTAFVVAG

GLUT10_HUMAN.O9 --------------------------LDFGLSCLEQEF-------------LVGSLLLGA

GLUT12_HUMAN.Q8 --------------------------TLLALSCHEQEM-------------VVSSLVIGA

DmSlc45.AAF5031 ---G------------------LSPLIGFFMSPLLGSISDRCKLRWGRRRPIISILSFGI

A.gambiae.EAA12 LFGAFVY------GYLVEKFGIKRTLQALVIPHSAFWIITY--------------LATSV

AgTRET1.AB36954 LAGGILG------GPMIEYLGRKNTILATATPFIISWLLIG--------------CATHV

AgGLUT.EAA11842 LAGGILG------GPMIEYLGRKNTILATATPFIISWLLIG--------------CATHV

TRET1_AEDAE.Q17 LAGGILG------GPLIEYLGRKNTILATATPFIISWLLIA--------------CATHV

PvTRET.BAF63703 LAGGIAG------GPFIEYLGRKNTILATAVPFIVAWLLIA--------------FANSI

DmTRET1-1A.AB36 LAGGIAG------GPLIEYLGRRNTILATAVPFIVSSLLIA--------------CAVNV

TRE11_DROME.A1Z LAGGIAG------GPLIEYLGRRNTILATAVPFIVSSLLIA--------------CAVNV

DmTret1-1B.AB36 LAGGIAG------GPLIEYLGRRNTILATAVPFIVSSLLIA--------------CAVNV

TRE11_DROME.A1Z LAGGIAG------GPLIEYLGRRNTILATAVPFIVSSLLIA--------------CAVNV

TRET1_DROPS.Q29 LAGGIAG------GPFIEYLGRRNTILATAIPFIVSSLLIA--------------CAVNV

DmTret1-2.AB369 LAGGITG------GPLIEYLGRRSTILATAVPFIVSSLLIA--------------CAVNV

TRE12_DROME.Q8M LAGGITG------GPLIEYLGRRSTILATAVPFIVSSLLIA--------------CAVNV

TRE12_DROME.Q8M LAGGITG------GPLIEYLGRRSTILATAVPFIVSSLLIA--------------CAVNV

NlST8TRET.BAI83 LFGGIAG------GPLIETIGRRTTILSTAIPFILSFLLIA--------------SATNV

AmTRET1.AB36954 LIGGIIG------GPCIEYIGRRNTILSTALPFLAGWLFIA--------------LATNV

SOLIN.Q2KKJ3 LVGGIAG------GPLIEYIGRKKTILVTAFPFIGAWLLIT--------------MAQNI

LOCMI.Q4VU77 LLGGVAG------GPLIEAIGRKTTILATAVPFIISFLLIG--------------LAVNV

BmTRET1.AB36955 LVGGIVG------GPLIEYLGRKKTIMGTAVPFTIGWMLIA--------------NAINV

NlST1GLUT.BAI83 MVGCLPM------SWMLDTFGRKSTIIILTVPTVAAWMMII--------------FAPSV

GLUT6_HUMAN.Q9U AAGGLSA------MILNDLLGRKLSIMFSAVPSAAGYALMA--------------GAHGL

GLUT6_HUMAN2.Q9 AAGGLSA------MILNDLLGRKLSIMFSAVPSAAGYALMA--------------GAHGL

GLUT8_HUMAN.Q9N AAGGVLG------GWLVDRAGRKLSLLLCSVPFVAGFAVIT--------------AAQDV

GLUT13_HUMAN.Q9 AVSALAG------GALNGVFGRRAAILLASALFTAGSAVLA--------------AANNK

GLUT1_HUMAN.P11 MIGSFSV------GLFVNRFGRRNSMLMMNLLAFVSAVLMG-----------FSKLGKSF

GLUT4_HUMAN.P14 MISSFLI------GIISQWLGRKRAMLVNNVLAVLGGSLMG-----------LANAAASY

GLUT3_HUMAN.P11 MIGSFSV------GLFVNRFGRRNSMLIVNLLAVTGGCFMG-----------LCKVAKSV

GLUT14_HUMAN.Q8 MIGSFSV------GLFVNRFGRRNSMLIVNLLAATGGCLMG-----------LCKIAESV

GLUT14_HUMAN2.Q MIGSFSV------GLFVNRFGRRNSMLIVNLLAATGGCLMG-----------LCKIAESV

GLUT2_HUMAN.P11 MTASFFG------GWLGDTLGRIKAMLVANILSLVGALLMG-----------FSKLGPSH

DmGlut1.Q6NNA9 MLGGFSG------GWMANRFGRKGGLLLNNVLGIAGACLMG-----------FTKVSHSY

DmGlut1B.Q8IRI6 MLGGFSG------GWMANRFGRKGGLLLNNVLGIAGACLMG-----------FTKVSHSY

GLUT5_HUMAN.P22 FIGSLLV------GPLVNKFGRKGALLFNNIFSIVPAILMG-----------CSRVATSF

GLUT9_HUMAN.Q9N LVGTLIV------KMIGKVLGRKHTLLANNGFAISAALLMA-----------CSLQAGAF

GLUT9_HUMAN2.Q9 LVGTLIV------KMIGKVLGRKHTLLANNGFAISAALLMA-----------CSLQAGAF

GLUT11_HUMAN.Q9 LFGALLA------GPLAITLGRKKSLLVNNIFVVSAAILFG-----------FSRKAGSF

GLUT11_HUMAN3.Q LFGALLA------GPLAITLGRKKSLLVNNIFVVSAAILFG-----------FSRKAGSF

GLUT11_HUMAN4.Q LFGALLA------GPLAITLGRKKSLLVNNIFVVSAAILFG-----------FSRKAGSF

SGTP1.AAA19731 AIGAFSC------GWVADGLGRRNGLILNNVIGIIGGVIVG-----------PCVLVKQP

SGTP4.AAA19733 AIGAFSC------GAIADCLGRRNGLIVNSLLAIIGGILVG-----------PCVAYSQP

GLUT10_HUMAN.O9 LLASLVG------GFLIDCYGRKQAILGSNLVLLAGSLTLG--------------LAGSL

GLUT12_HUMAN.Q8 LLASLTG------GVLIDRYGRRTAIILSSCLLGLGSLVLI--------------LSLSY

DmSlc45.AAF5031 MCGLILVPYGKDLGLLLGDAGYTYAESALNFTSSSGGSVAALVSGEATTGPSASDYKFAV

. . *

A.gambiae.EAA12 HQLYLARFLAGLSGGGIIVVFPLFIADISDKKIRGILGSFLALTSNSGFLLMYVIGDVLS

AgTRET1.AB36954 AMVLVGRALSGLCVGIASLSLPVYLGETVQPEVRGTLGLLPTAFGNIGILLCFVAGKYLD

AgGLUT.EAA11842 AMVLVGRALSGLCVGIASLSLPVYLGETVQPEVRGTLGLLPTAFGNIGILLCFVAGKYLD

TRET1_AEDAE.Q17 AMVLVGRALSGFSVGVASLSLPVYLGETVQPEVRGTLGLLPTAFGNIGILLCFVAGKYMD

PvTRET.BAF63703 WMVLAGRALSGFCVGIASLSLPVYLGETVQPEVRGTLGLLPTAFGNIGILICFVAGKYVN

DmTRET1-1A.AB36 AMVLCGRFLAGFCVGIASLSLPVYLGETVQPEVRGTLGLLPTAFGNIGILLCFVAGSFMN

TRE11_DROME.A1Z AMVLCGRFLAGFCVGIASLSLPVYLGETVQPEVRGTLGLLPTAFGNIGILLCFVAGSFMN

DmTret1-1B.AB36 AMVLCGRFLAGFCVGIASLSLPVYLGETVQPEVRGTLGLLPTAFGNIGILLCFVAGSFMN

TRE11_DROME.A1Z AMVLCGRFLAGFCVGIASLSLPVYLGETVQPEVRGTLGLLPTAFGNIGILLCFVAGSFMN

TRET1_DROPS.Q29 AMVLAGRFLAGFCVGIASLSLPVYLGETVQPEVRGTLGLLPTAFGNIGILLCFVAGTYMD

DmTret1-2.AB369 IMILCGRFLTGFCVGIASLSLPVYLGETLQPEVRGTLGLLPTALGNIGILVCYVAGSFMN

TRE12_DROME.Q8M IMILCGRFLTGFCVGIASLSLPVYLGETLQPEVRGTLGLLPTALGNIGILVCYVAGSFMN

TRE12_DROME.Q8M IMILCGRFLTGFCVGIASLSLPVYLGETLQPEVRGTLGLLPTALGNIGILVCYVAGSFMN

NlST8TRET.BAI83 ATILAGRSISGFCVGIASLALPVYLGETVQPEVRGTLGLLPTTFGNSGILICFIAGKYLD

AmTRET1.AB36954 AMILVGRSICGFCVGVASLSLPVYLGESIQPEVRGSLGLLPTVFGNSGILMCFTAGMYLA

SOLIN.Q2KKJ3 PMILAGRALCGFAVGVASLALPVCLGETIQAEVRGTLGLMPTVFGNTGILLCFVVGMYLD

LOCMI.Q4VU77 PMILAGRSVAGFCVGIASLCLPVYMGETVQAEVRGMLGLISTTFGNLGILLCYAIGNCLN

BmTRET1.AB36955 VMVFAGRVICGVCVGIVSLAFPVYIGETIQPEVRGALGLLPTAFGNTGILLAFLVGSYLD

NlST1GLUT.BAI83 TVICIARFILGFTTGAYAVAVPLYTSEISENEIRGTLGTYFQLQLTIGITSAYILGSLLP

GLUT6_HUMAN.Q9U WMLLLGRTLTGFAGGLTAACIPVYVSEIAPPGVRGALGATPQLMAVFGSLSLYALGLLLP

GLUT6_HUMAN2.Q9 WMLLLGRTLTGFAGGLTAACIPVYVSEIAPPGVRGALGATPQLMAVFGSLSLYALGLLLP

GLUT8_HUMAN.Q9N WMLLGGRLLTGLACGVASLVAPVYISEIAYPAVRGLLGSCVQLMVVVGILLAYLAGWVLE

GLUT13_HUMAN.Q9 ETLLAGRLVVGLGIGIASMTVPVYIAEVSPPNLRGRLVTINTLFITGGQFFASVVDGAFS

GLUT1_HUMAN.P11 EMLILGRFIIGVYCGLTTGFVPMYVGEVSPTALRGALGTLHQLGIVVGILIAQVFGLDSI

GLUT4_HUMAN.P14 EMLILGRFLIGAYSGLTSGLVPMYVGEIAPTHLRGALGTLNQLAIVIGILIAQVLGLESL

GLUT3_HUMAN.P11 EMLILGRLVIGLFCGLCTGFVPMYIGEISPTALRGAFGTLNQLGIVVGILVAQIFGLEFI

GLUT14_HUMAN.Q8 EMLILGRLVIGLFCGLCTGFVPMYIGEISPTALRGAFGTLNQLGIVIGILVAQIFGLELI

GLUT14_HUMAN2.Q EMLILGRLVIGLFCGLCTGFVPMYIGEISPTALRGAFGTLNQLGIVIGILVAQIFGLELI

GLUT2_HUMAN.P11 ILIIAGRSISGLYCGLISGLVPMYIGEIAPTALRGALGTFHQLAIVTGILISQIIGLEFI

DmGlut1.Q6NNA9 EMLFLGRFIIGVNCGLNTSLVPMYISEIAPLNLRGGLGTVNQLAVTVGLLLSQVLGIEQI

DmGlut1B.Q8IRI6 EMLFLGRFIIGVNCGLNTSLVPMYISEIAPLNLRGGLGTVNQLAVTVGLLLSQVLGIEQI

GLUT5_HUMAN.P22 ELIIISRLLVGICAGVSSNVVPMYLGELAPKNLRGALGVVPQLFITVGILVAQIFGLRNL

GLUT9_HUMAN.Q9N EMLIVGRFIMGIDGGVALSVLPMYLSEISPKEIRGSLGQVTAIFICIGVFTGQLLGLPEL

GLUT9_HUMAN2.Q9 EMLIVGRFIMGIDGGVALSVLPMYLSEISPKEIRGSLGQVTAIFICIGVFTGQLLGLPEL

GLUT11_HUMAN.Q9 EMIMLGRLLVGVNAGVSMNIQPMYLGESAPKELRGAVAMSSAIFTALGIVMGQVVGLREL

GLUT11_HUMAN3.Q EMIMLGRLLVGVNAGVSMNIQPMYLGESAPKELRGAVAMSSAIFTALGIVMGQVVGLREL

GLUT11_HUMAN4.Q EMIMLGRLLVGVNAGVSMNIQPMYLGESAPKELRGAVAMSSAIFTALGIVMGQVVGLREL

SGTP1.AAA19731 ALLYVGRFVIGINSGITIGIASLYLTEVAPRDLRGGIGACHQLAVTVGIAFSYFITFTFL

SGTP4.AAA19733 ALLFVGRVFNGFNFGISMGIAPMYLTEIAPLSLRGGIGSLHQLALTIGILVSYLMTLTYT

GLUT10_HUMAN.O9 AWLVLGRAVVGFAISLSSMACCIYVSELVGPRQRGVLVSLYEAGITVGILLSYALNYALA

GLUT12_HUMAN.Q8 TVLIVGRIAIGVSISLSSIATCVYIAEIAPQHRRGLLVSLNELMIVIGILSAYISNYAFA

DmSlc45.AAF5031 ILTILGMVLLDFDADTCQTPARTYLLDMCVPEEQPKAMTMFALFAGFGGTIGYAIGGVD-

. . . : : *

A.gambiae.EAA12 ------YH--------------TVALTMLALPLLFTVLMCFVPDTPQTC-LKKGR-----

AgTRET1.AB36954 ------WS--------------GLAFLGAALPIPFLLLMFLIPETPRWY-VSRNR-----

AgGLUT.EAA11842 ------WS--------------GLAFLGAALPIPFLLLMFLIPETPRWY-VSRNR-----

TRET1_AEDAE.Q17 ------WS--------------GLAFLGAALPIPFLLLMFLIPETPRWY-VSRGR-----

PvTRET.BAF63703 ------WS--------------GLAFIGSILPIPFMVLTLLIPETPRWF-VTRGR-----

DmTRET1-1A.AB36 ------WS--------------MLAFLGAALPVPFLILMFLIPETPRWF-VGRGL-----

TRE11_DROME.A1Z ------WS--------------MLAFLGAALPVPFLILMFLIPETPRWF-VGRGL-----

DmTret1-1B.AB36 ------WS--------------MLAFLGAALPVPFLILMFLIPETPRWF-VGRGL-----

TRE11_DROME.A1Z ------WS--------------MLAFLGAALPVPFLILMFLIPETPRWF-VGRGL-----

TRET1_DROPS.Q29 ------WS--------------MLAFLGAALPVPFLILMFLIPETPRWF-VSRGR-----

DmTret1-2.AB369 ------WS--------------MLAFLGAALPVPFLILMIIIPETPRWF-VNRGQ-----

TRE12_DROME.Q8M ------WS--------------MLAFLGAALPVPFLILMIIIPETPRWF-VNRGQ-----

TRE12_DROME.Q8M ------WS--------------MLAFLGAALPVPFLILMIIIPETPRWF-VNRGQ-----

NlST8TRET.BAI83 ------WS--------------LLAMLGAAIPVPFLLCMFLIPETPRWF-VEKGK-----

AmTRET1.AB36954 ------WR--------------NLALLGACIPIIFLILMFLIPETPRWY-ISKGK-----

SOLIN.Q2KKJ3 ------WR--------------NLALIGAILPLPFLILMFIIPETPRWY-ISKGK-----

LOCMI.Q4VU77 ------WW--------------KLALFGACLPVPFLVCTCFVPETPRWY-ISKNK-----

BmTRET1.AB36955 ------WS--------------NLAFFGAAIPVPFFLLMILTPETPRWY-VSKAR-----

NlST1GLUT.BAI83 ------IF--------------WMTMVCGCIPVVLALAMLIIPETPTYY-LKKFR-----

GLUT6_HUMAN.Q9U ------WR--------------WLAVAGEAPVLIMILLLSFMPNSPRFL-LSRGR-----

GLUT6_HUMAN2.Q9 ------WR--------------WLAVAGEAPVLIMILLLSFMPNSPRFL-LSRGR-----

GLUT8_HUMAN.Q9N ------WR--------------WLAVLGCVPPSLMLLLMCFMPETPRFL-LTQHR-----

GLUT13_HUMAN.Q9 YLQKDGWR--------------YMLGLAAVPAVIQFFGFLFLPESPRWL-IQKGQ-----

GLUT1_HUMAN.P11 MGNKDLWP--------------LLLSIIFIPALLQCIVLPFCPESPRFLLINRNE-----

GLUT4_HUMAN.P14 LGTASLWP--------------LLLGLTVLPALLQLVLLPFCPESPRYLYIIQNL-----

GLUT3_HUMAN.P11 LGSEELWP--------------LLLGFTILPAILQSAALPFCPESPRFLLINRKE-----

GLUT14_HUMAN.Q8 LGSEELWP--------------VLLGFTILPAILQSAALPCCPESPRFLLINRKK-----

GLUT14_HUMAN2.Q LGSEELWP--------------VLLGFTILPAILQSAALPCCPESPRFLLINRKK-----

GLUT2_HUMAN.P11 LGNYDLWH--------------ILLGLSGVRAILQSLLLFFCPESPRYLYIKLDE-----

DmGlut1.Q6NNA9 LGTNEGWP--------------ILLGLAICPAILQLILLPVCPESPRYLLITKQW-----

DmGlut1B.Q8IRI6 LGTNEGWP--------------ILLGLAICPAILQLILLPVCPESPRYLLITKQW-----

GLUT5_HUMAN.P22 LANVDGWP--------------ILLGLTGVPAALQLLLLPFFPESPRYLLIQKKD-----

GLUT9_HUMAN.Q9N LGKESTWP--------------YLFGVIVVPAVVQLLSLPFLPDSPRYLLLEKHN-----

GLUT9_HUMAN2.Q9 LGKESTWP--------------YLFGVIVVPAVVQLLSLPFLPDSPRYLLLEKHN-----

GLUT11_HUMAN.Q9 LGGPQAWP--------------LLLASCLVPGALQLASLPLLPESPRYLLIDCGD-----

GLUT11_HUMAN3.Q LGGPQAWP--------------LLLASCLVPGALQLASLPLLPESPRYLLIDCGD-----

GLUT11_HUMAN4.Q LGGPQAWP--------------LLLASCLVPGALQLASLPLLPESPRYLLIDCGD-----

SGTP1.AAA19731 LNTLNLWP--------------LAVALGAVPAAISLVTLPFCPESPRFLYMKKHK-----

SGTP4.AAA19733 LNTPTLWP--------------ISVAVGSVPALIALILLPYCPESPRFLFIKKGK-----

GLUT10_HUMAN.O9 -GTPWGWR--------------HMFGWATAPAVLQSLSLLFLPAGTD-------------

GLUT12_HUMAN.Q8 -NVFHGWK--------------YMFGLVIPLGVLQAIAMYFLPPSPRFL-VMKGQ-----

DmSlc45.AAF5031 ------WETTHIGSFMGGNIPTVFTLVTIIFAVCYLITVTTFREIPLPL-IEQDELLRPL

.

A.gambiae.EAA12 -TAEAERSFMFYRG------IRTQA--EKTSALRQEFDNMEKFI--------EHNSGQNS

AgTRET1.AB36954 -EDRARKALQWLRG--------------RKADVEPELKGISKSH-------QDAERHASS

AgGLUT.EAA11842 -EDRARKALQWLRG--------------RKADVEPELKGISKSH-------QDAERHASS

TRET1_AEDAE.Q17 -DDRARKALQWLRG--------------KKADVDPELKGIIKSH-------QDAERHASQ

PvTRET.BAF63703 -EERARKALQWLRG--------------KKADVEPELKGIVKSH-------CEAERHASQ

DmTRET1-1A.AB36 -EERARKALKWLRG--------------KEADVEPELKGLMRSQ-------ADADRQASR

TRE11_DROME.A1Z -EERARKALKWLRG--------------KEADVEPELKGLMRSQ-------ADADRQASR

DmTret1-1B.AB36 -EERARKALKWLRG--------------KEADVEPELKGLMRSQ-------ADADRQASR

TRE11_DROME.A1Z -EERARKALKWLRG--------------KEADVEPELKGLMRSQ-------ADADRQASR

TRET1_DROPS.Q29 -EEKARKALSWLRG--------------KEADVEPELKGLMRSQ-------ADADRQATQ

DmTret1-2.AB369 -EERARKALKWLRG--------------KEADVEPELKELMQSQ-------ADADRQATQ

TRE12_DROME.Q8M -EERARKALKWLRG--------------KEADVEPELKELMQSQ-------ADADRQATQ

TRE12_DROME.Q8M -EERARKALKWLRG--------------KEADVEPELKELMQSQ-------ADADRQATQ

NlST8TRET.BAI83 -QQRARKALQWLRG--------------NNTDVSYEFSEIEKSN-------KDAEKCENE

AmTRET1.AB36954 -IKEARKSLQWLRG--------------KTADISEELDSIQKMH-------IESERIATE

SOLIN.Q2KKJ3 -SKMSRKSLQWLRG--------------KDADITDELTMIEKLHQE----YLDSEQNASQ

LOCMI.Q4VU77 -TKRAHKALQWLRG--------------KDADVTAELHEIEKNH-------LDSIKNAPA

BmTRET1.AB36955 -VQEARKSLRWLRG--------------KNVNIEKEMRDLTISQ-------TESDR-TGG

NlST1GLUT.BAI83 -VDEARKALQWFRG--------------SHYDVEPELMLLKANL--------DQMEAERV

GLUT6_HUMAN.Q9U -DEEALRALAWLRG--------------TDVDVHWEFEQIQDNV-----------RRQSS

GLUT6_HUMAN2.Q9 -DEEALRALAWLRG--------------TDVDVHWEFEQIQDNV-----------RRQSS

GLUT8_HUMAN.Q9N -RQEAMAALRFLWG--------------SEQGWE------------------DPPIGAEQ

GLUT13_HUMAN.Q9 -TQKARRILSQMRG---------------NQTIDEEYDSIKNNIEE-----EEKEVGSAG

GLUT1_HUMAN.P11 -ENRAKSVLKKLRG---------------TADVTHDLQEMKEES--------RQMMREKK

GLUT4_HUMAN.P14 -EGPARKSLKRLTG---------------WADVSGVLAELKDEK--------RKLERERP

GLUT3_HUMAN.P11 -EENAKQILQRLWG---------------TQDVSQDIQEMKDES--------ARMSQEKQ

GLUT14_HUMAN.Q8 -EENATRILQRLWG---------------TQDVSQDIQEMKDES--------ARMSQEKQ

GLUT14_HUMAN2.Q -EENATRILQRLWG---------------TQDVSQDIQEMKDES--------ARMSQEKQ

GLUT2_HUMAN.P11 -EVKAKQSLKRLRG---------------YDDVTKDINEMRKER--------EEASSEQK

DmGlut1.Q6NNA9 -EEEARKALRRLRA---------------SGSVEEDIEEMRAEE--------RAQQSESH

DmGlut1B.Q8IRI6 -EEEARKALRRLRA---------------SGSVEEDIEEMRAEE--------RAQQSESH

GLUT5_HUMAN.P22 -EAAAKKALQTLRG---------------WDSVDREVAEIRQED--------EAEKAAGF

GLUT9_HUMAN.Q9N -EARAVKAFQTFLG---------------KADVSQEVEEVLAES--------RVQRSIRL

GLUT9_HUMAN2.Q9 -EARAVKAFQTFLG---------------KADVSQEVEEVLAES--------RVQRSIRL

GLUT11_HUMAN.Q9 -TEACLAALRRLRG---------------SGDLAGELEELEEER--------AACQGCRA

GLUT11_HUMAN3.Q -TEACLAALRRLRG---------------SGDLAGELEELEEER--------AACQGCRA

GLUT11_HUMAN4.Q -TEACLAALRRLRG---------------SGDLAGELEELEEER--------AACQGCRA

SGTP1.AAA19731 -EAEARKAFLQLNV---------------KENVDTFIGELREEI--------EVAKNQPV

SGTP4.AAA19733 -EAKARKAFQRLNC---------------IDDINETFNEMKREM--------HEAEKRPK

GLUT10_HUMAN.O9 -ETATHKDLIPLQG--------------GEAP--------------------KLGPGRPR

GLUT12_HUMAN.Q8 -EGAASKVLGRLRA---------------LSDTTEELTVIKSSL-----------KDEYQ

DmSlc45.AAF5031 SEQAIKKELKKKNNTIYYIQETTQLELQMASDDPKRLEALQGSYQNGYSPAVEKQGKSQD

:

A.gambiae.EAA12 RVTLADFKSR-EAKLGIFIGVFLM-----------FINQFCGIFAILTYA----ATIFAG

AgTRET1.AB36954 SAMLDLLNKA--NLKPLLISLGLM-----------FFQQLSGINAVIFYT----VQIFQS

AgGLUT.EAA11842 SAMLDLLNKA--NLKPLLISLGLM-----------FFQQLSGINAVIFYT----VQIFQS

TRET1_AEDAE.Q17 SAMLDLMKKA--NLKPLLISLGLM-----------FFQQLSGINAVIFYT----VQIFQD

PvTRET.BAF63703 NAIFDLMKRS--NLKPLLIALGLM-----------FFQQLSGINAVIFYT----VSIFKD

DmTRET1-1A.AB36 NTMLELLKLN--NLKPLSISLGLM-----------FFQQFSGINAVIFYT----VQIFKD

TRE11_DROME.A1Z NTMLELLKLN--NLKPLSISLGLM-----------FFQQFSGINAVIFYT----VQIFKD

DmTret1-1B.AB36 NTMLELLKLN--NLKPLSISLGLM-----------FFQQFSGINAVIFYT----VQIFKD

TRE11_DROME.A1Z NTMLELLKLN--NLKPLSISLGLM-----------FFQQFSGINAVIFYT----VQIFKD

TRET1_DROPS.Q29 NKMMELLKRN--NLKPLSISLGLM-----------FFQQLSGINAVIFYT----VSIFKD

DmTret1-2.AB369 NTCLELFKRN--NLKPLSISLGLM-----------FFQQFSGINAVIFYT----VQIFKD

TRE12_DROME.Q8M NTCLELFKRN--NLKPLSISLGLM-----------FFQQFSGINAVIFYT----VQIFKD

TRE12_DROME.Q8M NTCLELFKRN--NLKPLSISLGLM-----------FFQQFSGINAVIFYT----VQIFKD

NlST8TRET.BAI83 SAFKELFSAK--YSRPLIISIGLM-----------FFQQLSGINAVIFYT----VSIFKD

AmTRET1.AB36954 GALIELFRKN--HIKPVFISLGLM-----------FFQQFSGINAVIFYT----VQIFKD

SOLIN.Q2KKJ3 NMFSELTKSK--NLRPLLISLGLM-----------LFQQMSGINAVIFYT----VQIFQD

LOCMI.Q4VU77 SAL-DLFNRS--NIKPITVSMGLM-----------FFQQLSGINAVIFYT----VDIFRD

BmTRET1.AB36955 NAFKQLFSKR--YLPAVMISLGLM-----------LFQQLTGINAVIFYA----ASIFQM

NlST1GLUT.BAI83 PFTQAFVTTP--AKRGLVVGLGVM-----------FFQQFSGVNAVIFYA----ESIFKA

GLUT6_HUMAN.Q9U RVSWAEARAP-HVCRPITVALLMR-----------LLQQLTGITPILVYL----QSIFDS

GLUT6_HUMAN2.Q9 RVSWAEARAP-HVCRPITVALLMR-----------LLQQLTGITPILVYL----QSIFDS

GLUT8_HUMAN.Q9N SFHLALLRQP-GIYKPFIIGVSLM-----------AFQQLSGVNAVMFYA----ETIFEE

GLUT13_HUMAN.Q9 PVICRMLSYP-PTRRALIVGCGLQ-----------MFQQLSGINTIMYYS----ATILQM

GLUT1_HUMAN.P11 VTILELFRSP-AYRQPILIAVVLQ-----------LSQQLSGINAVFYYS----TSIFEK

GLUT4_HUMAN.P14 LSLLQLLGSR-THRQPLIIAVVLQ-----------LSQQLSGINAVFYYS----TSIFET

GLUT3_HUMAN.P11 VTVLELFRVS-SYRQPIIISIVLQ-----------LSQQLSGINAVFYYS----TGIFKD

GLUT14_HUMAN.Q8 VTVLELFRVS-SYRQPIIISIVLQ-----------LSQQLSGINAVFYYS----TGIFKD

GLUT14_HUMAN2.Q VTVLELFRVS-SYRQPIIISIVLQ-----------LSQQLSGINAVFYYS----TGIFKD

GLUT2_HUMAN.P11 VSIIQLFTNS-SYRQPILVALMLH-----------VAQQFSGINGIFYYS----TSIFQT

DmGlut1.Q6NNA9 ISTMELICSP-TLRPPLIIGIVMQ-----------LSQQFSGINAVFYYS----TSLFMS

DmGlut1B.Q8IRI6 ISTMELICSP-TLRPPLIIGIVMQ-----------LSQQFSGINAVFYYS----TSLFMS

GLUT5_HUMAN.P22 ISVLKLFRMR-SLRWQLLSIIVLM-----------GGQQLSGVNAIYYYA----DQIYLS

GLUT9_HUMAN.Q9N VSVLELLRAP-YVRWQVVTVIVTM-----------ACYQLCGLNAIWFYT----NSIFGK

GLUT9_HUMAN2.Q9 VSVLELLRAP-YVRWQVVTVIVTM-----------ACYQLCGLNAIWFYT----NSIFGK

GLUT11_HUMAN.Q9 RRPWELFQHR-ALRRQVTSLVVLG-----------SAMELCGNDSVYAYA----SSVFRK

GLUT11_HUMAN3.Q RRPWELFQHR-ALRRQVTSLVVLG-----------SAMELCGNDSVYAYA----SSVFRK

GLUT11_HUMAN4.Q RRPWELFQHR-ALRRQVTSLVVLG-----------SAMELCGNDSVYAYA----SSVFRK

SGTP1.AAA19731 FKFTQLFTQR-DLRMPVLIACLIQ-----------VLQQLSGINAVITYS----SLMLEL

SGTP4.AAA19733 FKFFRLFTQR-DLRMPVLIACIIQ-----------VFQQLSGINAVITYS----STMLKT

GLUT10_HUMAN.O9 YSFLDLFRARDNMRGRTTVGLGLV-----------LFQQLTGQPNVLCYA----STIFSS

GLUT12_HUMAN.Q8 YSFWDLFRSKDNMRTRIMIGLTLV-----------FFVQITGQPNILFYA----STVLKS

DmSlc45.AAF5031 LETQSDYDAP-VSLKAYLKSIFIMPYSMRMLALTNLFCWMGHVTYCLYFTDFVGEAVFHG

: : :

A.gambiae.EAA12 VGSIL--SPNTS-------------AIIMGTIQIVGTLSSFVFVDLAGRKVLLIISTFGT

AgTRET1.AB36954 AGSTI--DEKLC-------------TIIVGVVNFIATFIATVLIDRLGRKILLYISDVAM

AgGLUT.EAA11842 AGSTI--DEKLC-------------TIIVGVVNFIATFIATVLIDRLGRKILLYISDVAM

TRET1_AEDAE.Q17 AGSTI--DENLC-------------TIIVGVVNFIATFIATMLIDRLGRKMLLYISDVAM

PvTRET.BAF63703 AGSTI--DENLC-------------TIIVGVVNFGATFFATVLIDRLGRKILLYISEVAM

DmTRET1-1A.AB36 AGSTI--DGNLC-------------TIIVGIVNFLATFIGIVLIDRAGRKILLYVSDIAM

TRE11_DROME.A1Z AGSTI--DGNLC-------------TIIVGIVNFLATFIGIVLIDRAGRKILLYVSDIAM

DmTret1-1B.AB36 AGSTI--DGNLC-------------TIIVGIVNFLATFIGIVLIDRAGRKILLYVSDIAM

TRE11_DROME.A1Z AGSTI--DGNLC-------------TIIVGIVNFLATFIGIVLIDRAGRKILLYVSDIAM

TRET1_DROPS.Q29 AGSTI--DGNLC-------------TIIVGIVNFMATFIATLLIDRAGRKILLYVSNIAM

DmTret1-2.AB369 AGSTI--DSNLS-------------TIIVGVVNFFATFMGIILIDRLGRKILLYVSDIAM

TRE12_DROME.Q8M AGSTI--DSNLS-------------TIIVGVVNFFATFMGIILIDRLGRKILLYVSDIAM

TRE12_DROME.Q8M AGSTI--DSNLS-------------TIIVGVVNFFATFMGIILIDRLGRKILLYVSDIAM

NlST8TRET.BAI83 AGSTI--DENLS-------------TIIVGIVNMGSTFVATMLIDRLGRKILLYVSSTLM

AmTRET1.AB36954 SGSTV--DENLS-------------TIIVGLVNFISTFVAAMIIDRLGRKMLLYISSILM

SOLIN.Q2KKJ3 AGSTI--DENLS-------------TIIIGVVNFISTFVAASVIDKLGRKMLLYISAVLM

LOCMI.Q4VU77 AGSTI--DGNLS-------------TIIVGIVNLGSTFIATALIDRLGRKVLLYISAIAM

BmTRET1.AB36955 SGSSV--DENLA-------------SIIIGVVNFISTFIATMLIDRLGRKVLLYISSVAM

NlST1GLUT.BAI83 AGSSM--SPSLQ-------------TIIVGLIMVVMTWVATLAIDRAGRRPLLLISASIM

GLUT6_HUMAN.Q9U TAVLL--PPKDD-------------AAIVGAVRLLSVLIAALTMDLAGRKVLLFVSAAIM

GLUT6_HUMAN2.Q9 TAVLL--PPKDD-------------AAIVGAVRLLSVLIAALTMDLAGRKVLLFVS----

GLUT8_HUMAN.Q9N AKFK---DSSLA-------------SVVVGVIQVLFTAVAALIMDRAGRRLLLVLSGVVM

GLUT13_HUMAN.Q9 SGVED---DRLAI----------WLASVTAFTNFIFTLVGVWLVEKVGRRKLTFGSLAGT

GLUT1_HUMAN.P11 AGVQQ---PVYA-------------TIGSGIVNTAFTVVSLFVVERAGRRTLHLIGLAGM

GLUT4_HUMAN.P14 AGVGQ---PAYA-------------TIGAGVVNTVFTLVSVLLVERAGRRTLHLLGLAGM

GLUT3_HUMAN.P11 AGVQE---PIYA-------------TIGAGVVNTIFTVVSLFLVERAGRRTLHMIGLGGM

GLUT14_HUMAN.Q8 AGVQQ---PIYA-------------TISAGVVNTIFTLLSLFLVERAGRRTLHMIGLGGM

GLUT14_HUMAN2.Q AGVQQ---PIYA-------------TISAGVVNTIFTLLSLFLVERAGRRTLHMIGLGGM

GLUT2_HUMAN.P11 AGISK---PVYA-------------TIGVGAVNMVFTAVSVFLVEKAGRRSLFLIGMSGM

DmGlut1.Q6NNA9 SGLTE-ESAKFA-------------TIGIGAIMVVMTLVSIPLMDRTGRRTLHLYGLGGM

DmGlut1B.Q8IRI6 SGLTE-ESAKFA-------------TIGIGAIMVVMTLVSIPLMDRTGRRTLHLYGLGGM

GLUT5_HUMAN.P22 AGVPE-EHVQYV-------------TAGTGAVNVVMTFCAVFVVELLGRRLLLLLGFSIC

GLUT9_HUMAN.Q9N AGIPP-AKIPYV-------------TLSTGGIETLAAVFSGLVIEHLGRRPLLIGGFGLM

GLUT9_HUMAN2.Q9 AGIPP-AKIPYV-------------TLSTGGIETLAAVFSGLVIEHLGRRPLLIGGFGLM

GLUT11_HUMAN.Q9 AGVPE-AKIQYA-------------IIGTGSCELLTAVVSCVVIERVGRRVLLIGGYSLM

GLUT11_HUMAN3.Q AGVPE-AKIQYA-------------IIGTGSCELLTAVVSCVVIERVGRRVLLIGGYSLM

GLUT11_HUMAN4.Q AGVPE-AKIQYA-------------IIGTGSCELLTAVVSCVVIERVGRRVLLIGGYSLM

SGTP1.AAA19731 AGIPD-VYLQYC-------------VFAIGVLNVIVTVVSLPLIERAGRRTLLLWPTVSL

SGTP4.AAA19733 AGIPL-VYIQFC-------------VVAVPAINVLMTVLSVYLIERAGRRTLLLWPTVLL

GLUT10_HUMAN.O9 VGFHGGSSAVLA-------------SVGLGAVKVAATLTAMGLVDRAGRRALLLAGCALM

GLUT12_HUMAN.Q8 VGFQSNEAASLA-------------STGVGVVKVISTIPATLLVDHVGSKTFLCIGSSVM

DmSlc45.AAF5031 DPTAA-PNSEAALNYEAGVRFGCWGMAIYAFSCSIYSLSVTKLMKWFGTKAVYISGMIYY

:. * : .

A.gambiae.EAA12 GLGLFVLAVFNWLTVNMSTHW---------------------------------------

AgTRET1.AB36954 IITLMTLGTFFYMKN-----------------------------------NGDD------

AgGLUT.EAA11842 IITLMTLGTFFYMKN-----------------------------------NGDD------

TRET1_AEDAE.Q17 IITLMTLGGFFYVKN-----------------------------------SGQD------

PvTRET.BAF63703 VITLLTLGTFFYYKN-----------------------------------SGND------

DmTRET1-1A.AB36 VLTLFVLGGFFYCKT-----------------------------------YGPD------

TRE11_DROME.A1Z VLTLFVLGGFFYCKT-----------------------------------YGPD------

DmTret1-1B.AB36 VLTLFVLGGFFYCKT-----------------------------------YGPD------

TRE11_DROME.A1Z VLTLFVLGGFFYCKT-----------------------------------YGPD------

TRET1_DROPS.Q29 IITLFVLGGFFYCKS-----------------------------------HGQD------

DmTret1-2.AB369 IVTLSILGGFFYCKA-----------------------------------HGPD------

TRE12_DROME.Q8M IVTLSILGGFFYCKA-----------------------------------HGPD------

TRE12_DROME.Q8M IVTLSILGGFFYCKA-----------------------------------HGPD------

NlST8TRET.BAI83 TITLLILGTFFYVKN----------------------------------VMQID------

AmTRET1.AB36954 CITLFTFGTFFYVKE------------------------------------LMD------

SOLIN.Q2KKJ3 AVTLFSLGGFFYVKS-----------------------------------QDVD------

LOCMI.Q4VU77 NLSLLALGAFFFLKD-----------------------------------TGYD------

BmTRET1.AB36955 ITTLLALGAYFYLKQ-----------------------------------NHID------

NlST1GLUT.BAI83 AICTAILGVYFLLLE-----------------------------------KTPDF-----

GLUT6_HUMAN.Q9U FAANLTLGLYIHFGP--------RP-------------------------LSPNS-----

GLUT6_HUMAN2.Q9 ------------------------------------------------------------

GLUT8_HUMAN.Q9N VFSTSAFGAYFKLTQ-----------------------------------GGPG------

GLUT13_HUMAN.Q9 TVALIILALGFVLSAQVSPRITFKPIAPSGQNATCTRYSYCNE-----CMLDPDCGFCYK

GLUT1_HUMAN.P11 AGCAILMTIALALL----------------------------------------------

GLUT4_HUMAN.P14 CGCAILMTVALLLL----------------------------------------------

GLUT3_HUMAN.P11 AFCSTLMTVSLLLK----------------------------------------------

GLUT14_HUMAN.Q8 AFCSTLMTVSLLLK----------------------------------------------

GLUT14_HUMAN2.Q AFCSTLMTVSLLLK----------------------------------------------

GLUT2_HUMAN.P11 FVCAIFMSVGLVLL----------------------------------------------

DmGlut1.Q6NNA9 FIFSIFITISFLIKE---------------------FFGYVQ------------------

DmGlut1B.Q8IRI6 FIFSIFITISFLIK----------------------------------------------

GLUT5_HUMAN.P22 LIACCVLTAALALQ----------------------------------------------

GLUT9_HUMAN.Q9N GLFFGTLTITLTLQ----------------------------------------------

GLUT9_HUMAN2.Q9 GLFFGTLTITLTLQ----------------------------------------------

GLUT11_HUMAN.Q9 TCWGSIFTVALCLQ----------------------------------------------

GLUT11_HUMAN3.Q TCWGSIFTVALCLQ----------------------------------------------

GLUT11_HUMAN4.Q TCWGSIFTVALCLQ----------------------------------------------

SGTP1.AAA19731 ALSLLLLTIFVNLAD-----------------------------------SGPQ------

SGTP4.AAA19733 AFSLLCLTISVNIAS-----------------------------------STKDP-----

GLUT10_HUMAN.O9 ALSVSGIGLVSFAVPMD---------------------------------SGPSC---LA

GLUT12_HUMAN.Q8 AASLVTMGIVNLNIHMNFTHI-------------CRSHNSINQSLDESVIYGPGN---LS

DmSlc45.AAF5031 GIGMLVLGL---------------------------------------------------

A.gambiae.EAA12 ------------------------------------------------------------

AgTRET1.AB36954 ------------------------------------------------------------

AgGLUT.EAA11842 ------------------------------------------------------------

TRET1_AEDAE.Q17 ------------------------------------------------------------

PvTRET.BAF63703 ------------------------------------------------------------

DmTRET1-1A.AB36 ------------------------------------------------------------

TRE11_DROME.A1Z ------------------------------------------------------------

DmTret1-1B.AB36 ------------------------------------------------------------

TRE11_DROME.A1Z ------------------------------------------------------------

TRET1_DROPS.Q29 ------------------------------------------------------------

DmTret1-2.AB369 ------------------------------------------------------------

TRE12_DROME.Q8M ------------------------------------------------------------

TRE12_DROME.Q8M ------------------------------------------------------------

NlST8TRET.BAI83 ------------------------------------------------------------

AmTRET1.AB36954 ------------------------------------------------------------

SOLIN.Q2KKJ3 ------------------------------------------------------------

LOCMI.Q4VU77 ------------------------------------------------------------

BmTRET1.AB36955 ------------------------------------------------------------

NlST1GLUT.BAI83 ------------------------------------------------------------

GLUT6_HUMAN.Q9U ----------------------------------------TAGLESESWGDLAQPLAAP-

GLUT6_HUMAN2.Q9 ------------------------------------------------------------

GLUT8_HUMAN.Q9N -----------------NSSHVAI-----------------------SAPVSAQPVDA--

GLUT13_HUMAN.Q9 MNKST----------VIDSSCVPVN----------KASTNEAAWGRCENETKFKTEDIF-

GLUT1_HUMAN.P11 --------------------------------------------------------EQ--

GLUT4_HUMAN.P14 --------------------------------------------------------ER--

GLUT3_HUMAN.P11 --------------------------------------------------------DN--

GLUT14_HUMAN.Q8 --------------------------------------------------------NH--

GLUT14_HUMAN2.Q --------------------------------------------------------NH--

GLUT2_HUMAN.P11 --------------------------------------------------------NK--

DmGlut1.Q6NNA9 --------------------------------------------------------EM--

DmGlut1B.Q8IRI6 --------------------------------------------------------EM--

GLUT5_HUMAN.P22 --------------------------------------------------------DT--

GLUT9_HUMAN.Q9N --------------------------------------------------------DH--

GLUT9_HUMAN2.Q9 --------------------------------------------------------DH--

GLUT11_HUMAN.Q9 --------------------------------------------------------SS--

GLUT11_HUMAN3.Q --------------------------------------------------------SS--

GLUT11_HUMAN4.Q --------------------------------------------------------SS--

SGTP1.AAA19731 --------------------------------------------------------ST--

SGTP4.AAA19733 --------------------------------------------------------TT--

GLUT10_HUMAN.O9 VPNATGQTGLPGDSGLLQDSSLP---PIPRTNEDQREPILSTAKKTKPHPRSGDPSAPPR

GLUT12_HUMAN.Q8 TNNNTLRDHFKGISSHSRSSLMPLRNDVDKRGETTSASLLNAGLSHTEYQIVTDPGDV--

DmSlc45.AAF5031 ------------------------------------------------------------

A.gambiae.EAA12 ----------------IQDYSWFPIVSLSATVYLFSIGLC---SIPFFVLP---------

AgTRET1.AB36954 ----------------VSEIGWLPLAAFVVFVVGFSLGFG---PIPWLMMG---------

AgGLUT.EAA11842 ----------------VSEIGWLPLAAFVVFVVGFSLGFG---PIPWLMMG---------

TRET1_AEDAE.Q17 ----------------VSQVGWLPLAAFVIYVLGFSLGFG---PIPWLMMG---------

PvTRET.BAF63703 ----------------VSNIGWLPLASFVIYVIGFSSGVG---PIPWLMLG---------

DmTRET1-1A.AB36 ----------------VSHLGWLPLTCFVIYILGFSLGFG---PIPWLMMG---------

TRE11_DROME.A1Z ----------------VSHLGWLPLTCFVIYILGFSLGFG---PIPWLMMG---------

DmTret1-1B.AB36 ----------------VSHLGWLPLTCFVIYILGFSLGFG---PIPWLMMG---------

TRE11_DROME.A1Z ----------------VSHLGWLPLTCFVIYILGFSLGFG---PIPWLMMG---------

TRET1_DROPS.Q29 ----------------VSQLGWLPLSCFVIYILGFSLGFG---PIPWLMMG---------

DmTret1-2.AB369 ----------------VSHLGWLPLTCFVIYILGFSLGFG---PIPWLMMG---------

TRE12_DROME.Q8M ----------------VSHLGWLPLTCFVIYILGFSLGFG---PIPWLMMG---------

TRE12_DROME.Q8M ----------------VSHLGWLPLTCFVIYILGFSLGFG---PIPWLMMG---------

NlST8TRET.BAI83 ----------------TTEYGWVPLGSFVVFVIGFSIGFG---PIPWLMLG---------

AmTRET1.AB36954 ----------------VTAFGWIPLMSLIVYVIGFSFGFG---PIPWLMMG---------

SOLIN.Q2KKJ3 ----------------VTAFGWLPLVSLIVYVIGFSLGFG---PIPWLMMG---------

LOCMI.Q4VU77 ----------------VQEYGWLPLASFVIFVVGFSLGFG---PIPWLMMG---------

BmTRET1.AB36955 ----------------VTAYGWLPLACLVIYVLGFSIGFG---PIPWLMLG---------

NlST1GLUT.BAI83 ----------------AKTIGSVPIVSLSIFIIVFSLGFG---PIPWMFMS---------

GLUT6_HUMAN.Q9U ----------------AGYLTLVPLLATMLFIMGYAVGWG---PITWLLMS---------

GLUT6_HUMAN2.Q9 ---------------------------------GYAVGWG---PITWLLMS---------

GLUT8_HUMAN.Q9N ----------------SVGLAWLAVGSMCLFIAGFAVGWG---PIPWLLMS---------

GLUT13_HUMAN.Q9 WAYNFC----------PTPYSWTALLGLILYLVFFAPGMG---PMPWTVNS---------

GLUT1_HUMAN.P11 ----------------LPWMSYLSIVAIFGFVAFFEVGPG---PIPWFIVA---------

GLUT4_HUMAN.P14 ----------------VPAMSYVSIVAIFGFVAFFEIGPG---PIPWFIVA---------

GLUT3_HUMAN.P11 ----------------YNGMSFVCIGAILVFVAFFEIGPG---PIPWFIVA---------

GLUT14_HUMAN.Q8 ----------------YNGMSFVCIGAILVFVACFEIGPG---PIPWFIVA---------

GLUT14_HUMAN2.Q ----------------YNGMSFVCIGAILVFVACFEIGPG---PIPWFIVA---------

GLUT2_HUMAN.P11 ----------------FSWMSYVSMIAIFLFVSFFEIGPG---PIPWFMVA---------

DmGlut1.Q6NNA9 ----------------IDWMSYLSVVATLGFVVFFAVGPG---SIPWMITA---------

DmGlut1B.Q8IRI6 ----------------IDWMSYLSVVATLGFVVFFAVGPG---SIPWMITA---------

GLUT5_HUMAN.P22 ----------------VSWMPYISIVCVISYVIGHALGPS---PIPALLIT---------

GLUT9_HUMAN.Q9N ----------------APWVPYLSIVGILAIIASFCSGPG---GIPFILTG---------

GLUT9_HUMAN2.Q9 ----------------APWVPYLSIVGILAIIASFCSGPG---GIPFILTG---------

GLUT11_HUMAN.Q9 ----------------FPWTLYLAMACIFAFILSFGIGPA---GVTGILAT---------

GLUT11_HUMAN3.Q ----------------FPWTLYLAMACIFAFILSFGIGPA---GVTGILAT---------

GLUT11_HUMAN4.Q ----------------FPWTLYLAMACIFAFILSFGIGPA---GVTGILAT---------

SGTP1.AAA19731 ----------------KNAMGIISIILILIYICSFALGLG---PVPALIVS---------

SGTP4.AAA19733 ----------------ARTAGIISAVLIILYICGFALGLG---PIPGVIVA---------

GLUT10_HUMAN.O9 LALSSALPGPPLPARGHALLRWTALLCLMVFVSAFSFGFG---PVTWLVLS---------

GLUT12_HUMAN.Q8 ----------------PAFLKWLSLASLLVYVAAFSIGLG---PMPWLVLS---------

DmSlc45.AAF5031 ---------------------WPTKWGVLVFSTSAGILYGTIFTVPFILVARYHAKNCFS

:. .

A.gambiae.EAA12 ----ELLPLKIC---NAGNTLSMVSITIFAFISLKIFPIMVEVINIYGVLGLYAGI----

AgTRET1.AB36954 ----EILPGKIR---GSAASVATAFNWSCTFVVTKTFADITASIGNHGAFWMFGSI----

AgGLUT.EAA11842 ----EILPGKIR---GSAASVATAFNWSCTFVVTKTFADITASIGNHGAFWMFGSI----

TRET1_AEDAE.Q17 ----EILPGKIR---GSAASVATAFNWSCTFIVTKTFADIINAIGTHGTFWMFGSI----

PvTRET.BAF63703 ----EILPGKIR---GSAASVATGFNWTCTFIVTKTFADIVAAIGNHGAFWFFGVI----

DmTRET1-1A.AB36 ----EILPAKIR---GSAASVATAFNWFCTFVVTKTFQDLTVAMGAHGAFWLFGAI----

TRE11_DROME.A1Z ----EILPAKIR---GSAASVATAFNWFCTFVVTKTFQDLTVAMGAHGAFWLFGAI----

DmTret1-1B.AB36 ----EILPAKIR---GSAASVATAFNWFCTFVVTKTFQDLTVAMGAHGAFWLFGAI----

TRE11_DROME.A1Z ----EILPAKIR---GSAASVATAFNWFCTFVVTKTFQDLTVAMGAHGAFWLFGAI----

TRET1_DROPS.Q29 ----EILPSKIR---GSAASVATAFNWSCTFVVTKTFQDMIDFMGAHGAFWLFGSI----

DmTret1-2.AB369 ----EILPAKIR---GPAASVVTAFNWFCTFVVTKTFQDLTVAMGAHGAFWLFGAI----

TRE12_DROME.Q8M ----EILPAKIR---GPAASVVTAFNWFCTFVVTKTFQDLTVAMGAHGAFWLFGAI----

TRE12_DROME.Q8M ----EILPAKIR---GPAASVVTAFNWFCTFVVTKTFQDLTVAMGAHGAFWLFGAI----

NlST8TRET.BAI83 ----EILPAKIR---GTAAALATGFNWSCTFLVTKSFSDLKAILGQHGAFWMFGVI----

AmTRET1.AB36954 ----EILPVKIR---GTAASVATAFNWSCTFVVTKTYEDLVLHIGPYGTFWLFGTL----

SOLIN.Q2KKJ3 ----EILPANIR---GSAASIATSFNWLCTFIVTKTFEDVIGVIGTHGTFWMFGII----

LOCMI.Q4VU77 ----EILPAKIR---GPAASVATAFNWSCTFIVTKTFSDLKGAVGPYGAFWIFSAI----

BmTRET1.AB36955 ----EILPSKIR---GTAASLATGFNWTCTFIVTKTFQNIIDAIYMHGTLWLFAVI----

NlST1GLUT.BAI83 ----EIFPPQIK---GPACSIACFFNWFSVFMVTKFFGDLQSKFGSYGTFWIFSGI----

GLUT6_HUMAN.Q9U ----EVLPLRAR---GVASGLCVLASWLTAFVLTKSFLPVVSTFGLQVPFFFFAAI----

GLUT6_HUMAN2.Q9 ----EVLPLRAR---GVASGLCVLASWLTAFVLTKSFLPVVSTFGLQVPFFFFAAI----

GLUT8_HUMAN.Q9N ----EIFPLHVK---GVATGICVLTNWLMAFLVTKEFSSLMEVLRPYGAFWLASAF----

GLUT13_HUMAN.Q9 ----EIYPLWAR---STGNACSSGINWIFNVLVSLTFLHTAEYLTYYGAFFLYAGF----

GLUT1_HUMAN.P11 ----ELFSQGPR---PAAIAVAGFSNWTSNFIVGMCFQYVEQLCGPY-VFIIFTVL----

GLUT4_HUMAN.P14 ----ELFSQGPR---PAAMAVAGFSNWTSNFIIGMGFQYVAEAMGPY-VFLLFAVL----

GLUT3_HUMAN.P11 ----ELFSQGPR---PAAMAVAGCSNWTSNFLVGLLFPSAAHYLGAY-VFIIFTGF----

GLUT14_HUMAN.Q8 ----ELFSQGPR---PAAMAVAGCSNWTSNFLVGLLFPSAAYYLGAY-VFIIFTGF----

GLUT14_HUMAN2.Q ----ELFSQGPR---PAAMAVAGCSNWTSNFLVGLLFPSAAYYLGAY-VFIIFTGF----

GLUT2_HUMAN.P11 ----EFFSQGPR---PAALAIAAFSNWTCNFIVALCFQYIADFCGPY-VFFLFAGV----

DmGlut1.Q6NNA9 ----ELFSQGPR---PSAMAIAVLVNWMANFVVGIGFPSMKTALENY-TFLPFSVF----

DmGlut1B.Q8IRI6 ----ELFSQGPR---PSAMAIAVLVNWMANFVVGIGFPSMKTALENY-TFLPFSVF----

GLUT5_HUMAN.P22 ----EIFLQSSR---PSAFMVGGSVHWLSNFTVGLIFPFIQEGLGPY-SFIVFAVI----

GLUT9_HUMAN.Q9N ----EFFQQSQR---PAAFIIAGTVNWLSNFAVGLLFPFIQKSLDTY-CFLVFATI----

GLUT9_HUMAN2.Q9 ----EFFQQSQR---PAAFIIAGTVNWLSNFAVGLLFPFIQKSLDTY-CFLVFATI----

GLUT11_HUMAN.Q9 ----ELFDQMAR---PAACMVCGALMWIMLILVGLGFPFIMEALSHF-LYVPFLGV----

GLUT11_HUMAN3.Q ----ELFDQMAR---PAACMVCGALMWIMLILVGLGFPFIMEALSHF-LYVPFLGV----

GLUT11_HUMAN4.Q ----ELFDQMAR---PAACMVCGALMWIMLILVGLGFPFIMEALSHF-LYVPFLGV----

SGTP1.AAA19731 ----EIFRQGPR---AAAYSLSQSIQWLSNLIVLCSYPVIQKNIGGY-SFLPFLVV----

SGTP4.AAA19733 ----EIFRQEPR---AAAYSLSQGVNLLCNLLVLFSYPSINDAIGGY-SFLPFLVI----

GLUT10_HUMAN.O9 ----EIYPVEIR---GRAFAFCNSFNWAANLFISLSFLDLIGTIGLSWTFLLYGLT----

GLUT12_HUMAN.Q8 ----EIFPGGIR---GRAMALTSSMNWGINLLISLTFLTVTDLIGLPWVCFIYTIM----

DmSlc45.AAF5031 IKNGEIVPLKQARGLGTDVAIISSMVFIAQLIVSLSVGPLVSWMDTTCAVLYASTFLSFL

*. .

A.gambiae.EAA12 SFAGVAVITFIVPETKGKNLISPQSV----------------------------------

AgTRET1.AB36954 CIVGLLFVIVYVPETQGKSLEDIERKMMGR------------------------------

AgGLUT.EAA11842 CIVGLLFVIVYVPETQGKSLEDIERKMMGR------------------------------

TRET1_AEDAE.Q17 CVIGLAFVIFYVPETQGKSLEDIERKMMGR------------------------------

PvTRET.BAF63703 CLIGLFFVIFFVPETQGKSLEEIERKMMGR------------------------------

DmTRET1-1A.AB36 CFVGLFFVIIYVPETQGKTLEDIERKMMGR------------------------------

TRE11_DROME.A1Z CFVGLFFVIIYVPETQGKTLEDIERKMMGR------------------------------

DmTret1-1B.AB36 CFVGLFFVIIYVPETQGKTLEDIERKMMGR------------------------------

TRE11_DROME.A1Z CFVGLFFVIIYVPETQGKTLEDIERKMMGR------------------------------

TRET1_DROPS.Q29 CFIGLFFVILYVPETQGKTLEDIERKMMGR------------------------------

DmTret1-2.AB369 CIVGLFFVIIFVPETRGKSLEEIERKMMGR------------------------------

TRE12_DROME.Q8M CIVGLFFVIIFVPETRGKSLEEIERKMMGR------------------------------

TRE12_DROME.Q8M CIVGLFFVIIFVPETRGKSLEEIERKMMGR------------------------------

NlST8TRET.BAI83 CLFGLVFVILLVPETQGKSLEDIERNLTGSGKDK-------------VPV----------

AmTRET1.AB36954 VAVAFIFVIICVPETRGRSLEEIERRFAGP------------------------------

SOLIN.Q2KKJ3 VVMGFVFVIISVPETRGRSLEEIEKKFTGP------------------------------

LOCMI.Q4VU77 CFFSLIFVKFCVPETQGKSLEDIERKFNGP------------------------------

BmTRET1.AB36955 CIGGLLFVIFFVPETKGKSLEEIEMKLTSG------------------------------

NlST1GLUT.BAI83 SIAGTFFVLNLVPETKGKSMEEIQKELGAT------------------P-----------

GLUT6_HUMAN.Q9U CLVSLVFTGCCVPETKGRSLEQIESFFRTGRRS-----FLR-------------------

GLUT6_HUMAN2.Q9 CLVSLVFTGCCVPETKGRSLEQIESFFRTGRRS-----FLR-------------------

GLUT8_HUMAN.Q9N CIFSVLFTLFCVPETKGKTLEQITAHFEGR------------------------------

GLUT13_HUMAN.Q9 AAVGLLFIYGCLPETKGKKLEEIESLFDNRLCT------------------CGT--SDSD

GLUT1_HUMAN.P11 LVLFFIFTYFKVPETKGRTFDEIASGFRQGGAS------------------QSDKTPEEL

GLUT4_HUMAN.P14 LLGFFIFTFLRVPETRGRTFDQISAAFHRTPSLL-----------------EQEVKPSTE

GLUT3_HUMAN.P11 LITFLAFTFFKVPETRGRTFEDITRAFEGQAHG-------------------ADRSGKDG

GLUT14_HUMAN.Q8 LITFLAFTFFKVPETRGRTFEDITRAFEGQAHG-------------------ADRSGKDG

GLUT14_HUMAN2.Q LITFLAFTFFKVPETRGRTFEDITRAFEGQAHG-------------------ADRSGKDG

GLUT2_HUMAN.P11 LLAFTLFTFFKVPETKGKSFEEIAAEFQKKSGS------------------AHRPKAAVE

DmGlut1.Q6NNA9 LAIFWIFTYKKVPETKNKTFEEILALFRHNNGR------------------YVSLH----

DmGlut1B.Q8IRI6 LAIFWIFTYKKVPETKNKTFEEILALFRHNNGR------------------YVSLH----

GLUT5_HUMAN.P22 CLLTTIYIFLIVPETKAKTFIEINQIFTKMNKV------------------SEVYPEKEE

GLUT9_HUMAN.Q9N CITGAIYLYFVLPETKNRTYAEISQAFSKRNKA---------------------YPPEEK

GLUT9_HUMAN2.Q9 CITGAIYLYFVLPETKNRTYAEISQAFSKRNKA---------------------YPPEEK

GLUT11_HUMAN.Q9 CVCGAIYTGLFLPETKGKTFQEISKELHRLN-----------------------FPRRAQ

GLUT11_HUMAN3.Q CVCGAIYTGLFLPETKGKTFQEISKELHRLN-----------------------FPRRAQ

GLUT11_HUMAN4.Q CVCGAIYTGLFLPETKGKTFQEISKELHRLN-----------------------FPRRAQ

SGTP1.AAA19731 VVICWIFFFLFMPETKNRTFDEVARDLAFGNIV------------------VGKRTTALE

SGTP4.AAA19733 VIICWIFFFLYMIETKNRTCDSNARDLATAKVV------------------ACQRPSRLT

GLUT10_HUMAN.O9 AVLGLGFIYLFVPETKGQSLAEIDQQFQKRRFT---LSF-----------------GHRQ

GLUT12_HUMAN.Q8 SLASLLFVVMFIPETKGCSLEQISMELAKVNYVKNNICFMSHHQEELVPKQPQKRKPQEQ

DmSlc45.AAF5031 AAIAAMFVLYV-------------------------------------------------

A.gambiae.EAA12 ----------------------------------------------

AgTRET1.AB36954 ------------VRRMSSVANIKPLSFNM-----------------

AgGLUT.EAA11842 ------------VRRMSSVANIKPLSFNM-----------------

TRET1_AEDAE.Q17 ------------VRRMSSVANIKPLSFNM-----------------

PvTRET.BAF63703 ------------VRRMSSVANMKPLSFNM-----------------

DmTRET1-1A.AB36 ------------VRRMSSVANIKPLSFNM-----------------

TRE11_DROME.A1Z ------------VRRMSSVANIKPLSFNM-----------------

DmTret1-1B.AB36 ------------VRRMSSVANIKPLSFNM-----------------

TRE11_DROME.A1Z ------------VRRMSSVANIKPLSFNM-----------------

TRET1_DROPS.Q29 ------------VRRMSSVANMKPLAFNM-----------------

DmTret1-2.AB369 ------------V-PMSSVVNIKPFPFNM-----------------

TRE12_DROME.Q8M ------------V-PMSSVVNIKPFPFNM-----------------

TRE12_DROME.Q8M ------------V-PMSSVVNIKPFPFNM-----------------

NlST8TRET.BAI83 ----------RTVRRMSSIANLKPLPSSI-----------------

AmTRET1.AB36954 ------------VRRTSAIANLKPMPITI-----------------

SOLIN.Q2KKJ3 ------------VRRMSAVANMKPTPMSC-----------------

LOCMI.Q4VU77 ------------VRRMSSIANLKPMPMAV-----------------

BmTRET1.AB36955 ------------SRRVRNISKQ---PENI----------------C

NlST1GLUT.BAI83 --------------QMTPEDRMENGQKPAKF---------------

GLUT6_HUMAN.Q9U ----------------------------------------------

GLUT6_HUMAN2.Q9 ----------------------------------------------

GLUT8_HUMAN.Q9N ----------------------------------------------

GLUT13_HUMAN.Q9 ------------EGRYIEYIRVKGSNYHLS--------DNDASDVE

GLUT1_HUMAN.P11 ------------FHPLGADSQV------------------------

GLUT4_HUMAN.P14 ------------LEYLGPDEND------------------------

GLUT3_HUMAN.P11 VME---------MNSIEPAKETTTNV--------------------

GLUT14_HUMAN.Q8 VMG---------MNSIEPAKETTTNV--------------------

GLUT14_HUMAN2.Q VMG---------MNSIEPAKETTTNV--------------------

GLUT2_HUMAN.P11 ------------MKFLGATETV------------------------

DmGlut1.Q6NNA9 ----------------------------------------------

DmGlut1B.Q8IRI6 ----------------------------------------------

GLUT5_HUMAN.P22 ------------LKELPPVTSEQ-----------------------

GLUT9_HUMAN.Q9N ------------IDSAVTDGKINGRP--------------------

GLUT9_HUMAN2.Q9 ------------IDSAVTDGKINGRP--------------------

GLUT11_HUMAN.Q9 G---------PTWRSLEVIQSTEL----------------------

GLUT11_HUMAN3.Q G---------PTWRSLEVIQSTEL----------------------

GLUT11_HUMAN4.Q G---------PTWRSLEVIQSTEL----------------------

SGTP1.AAA19731 ------------DRNLTVFTKQGNNEGPASESLLYPRSDNDKGMYA

SGTP4.AAA19733 ------------YKNEEPFYSDE-----------------------

GLUT10_HUMAN.O9 ------------NSTGIPYSRIEISAAS------------------

GLUT12_HUMAN.Q8 LLECNKLCGRGQSRQLSPET--------------------------

DmSlc45.AAF5031 ----------------------------------------------
